# Metabotypes of flavan-3-ol colonic metabolites after cranberry intake: elucidation and statistical approaches

**DOI:** 10.1007/s00394-021-02692-z

**Published:** 2021-11-09

**Authors:** Pedro Mena, Claudia Favari, Animesh Acharjee, Saisakul Chernbumroong, Letizia Bresciani, Claudio Curti, Furio Brighenti, Christian Heiss, Ana Rodriguez-Mateos, Daniele Del Rio

**Affiliations:** 1 Human Nutrition Unit, Department of Food & Drug, University of Parma, Via Volturno 39, 43125 Parma, Italy; 2 College of Medical and Dental Sciences, Institute of Cancer and Genomic Sciences, Centre for Computational Biology, University of Birmingham, Birmingham, B15 2TT UK; 3 Institute of Translational Medicine, University Hospitals Birmingham NHS, Foundation Trust, Birmingham, B15 2TT UK; 4 NIHR Surgical Reconstruction and Microbiology Research Centre, University Hospital Birmingham, Birmingham, B15 2WB UK; 5 Department of Food & Drug, University of Parma, Parma, Italy; 6 Department of Clinical and Experimental Medicine, Faculty of Health and Medical Sciences, University of Surrey, Guildford, UK; 7 Department of Nutritional Sciences, Faculty of Life Sciences and Medicine, King’s College London, London, UK; 8 School of Advanced Studies On Food and Nutrition, University of Parma, Parma, Italy

**Keywords:** Metabotypes, Flavan-3-ols, Inter-individual variation, Phenolic metabolites, Phenyl-γ-valerolactones

## Abstract

**Purpose:**

Extensive inter-individual variability exists in the production of flavan-3-ol metabolites. Preliminary metabolic phenotypes (metabotypes) have been defined, but there is no consensus on the existence of metabotypes associated with the catabolism of catechins and proanthocyanidins. This study aims at elucidating the presence of different metabotypes in the urinary excretion of main flavan-3-ol colonic metabolites after consumption of cranberry products and at assessing the impact of the statistical technique used for metabotyping.

**Methods:**

Data on urinary concentrations of phenyl-γ-valerolactones and 3-(hydroxyphenyl)propanoic acid derivatives from two human interventions has been used. Different multivariate statistics, principal component analysis (PCA), cluster analysis, and partial least square-discriminant analysis (PLS-DA), have been considered.

**Results:**

Data pre-treatment plays a major role on resulting PCA models. Cluster analysis based on k-means and a final consensus algorithm lead to quantitative-based models, while the expectation–maximization algorithm and clustering according to principal component scores yield metabotypes characterized by quali-quantitative differences in the excretion of colonic metabolites. PLS-DA, together with univariate analyses, has served to validate the urinary metabotypes in the production of flavan-3-ol metabolites and to confirm the robustness of the methodological approach.

**Conclusions:**

This work proposes a methodological workflow for metabotype definition and highlights the importance of data pre-treatment and clustering methods on the final outcomes for a given dataset. It represents an additional step toward the understanding of the inter-individual variability in flavan-3-ol metabolism.

**Trial registration:**

The acute study was registered at clinicaltrials.gov as NCT02517775, August 7, 2015; the chronic study was registered at clinicaltrials.gov as NCT02764749, May 6, 2016.

**Supplementary Information:**

The online version contains supplementary material available at 10.1007/s00394-021-02692-z.

## Introduction

Flavan-3-ols are characteristic polyphenols of tea, cocoa, wine, pome fruits (as apple and pear), berries, and nuts, but they are also found in stone fruits and legumes [1, 2]. This subclass of compounds is the main dietary source of flavonoids in Western diets [3–5] and has been associated with beneficial effects on the prevention of cardiometabolic diseases [6–9]. In addition, other putative benefits have been observed against cognitive decline [10, 11] and urinary tract infections [12, 13]. In plant-based foods, they occur as simple monomers or as oligomers and polymers of up to 190 units (also known as proanthocyanidins or condensed tannins) [14]. When ingested, both monomeric and high molecular weight flavan-3-ols are poorly absorbed and metabolized in the first gastrointestinal tract, reaching the colon and becoming a suitable substrate for the local microbiota [15]. These compounds undergo an extensive microbial metabolism leading to the formation of specific metabolites, namely phenyl-γ-valerolactones (PVLs) and phenylvaleric acids (PVAs), as well as of common end-products of (poly)phenol colonic catabolism, such as phenylpropanoic, phenylacetic, and benzoic acid derivatives [16–19]. The microbial metabolites are then absorbed by colonocytes before reaching the liver and are converted into phase II conjugated derivatives. Conjugated PVLs (sulfate, glucuronide, methoxy, and combinations thereof) are the main colonic circulating metabolites after ingestion of monomeric and polymeric flavan-3-ols by humans [19–23] and, in particular, sulfate and glucuronide derivatives of 5-(3ʹ,4ʹ-dihydroxyphenyl)-γ-valerolactone have been proposed as biomarkers of flavan-3-ol intake [23, 24]. These metabolites may be responsible for the health effects attributed to flavan-3-ols, as they are circulating molecules potentially available to target tissues and organs prior to be excreted in urine [19].

An extensive inter-individual variability is reported in the production of flavan-3-ol metabolites [20–23, 25–29], possibly affecting, at individual level, the health benefits associated with this class of compounds [19]. This variability might be due to personal differences in gut microbiota composition, resulting in different metabolic phenotypes or metabotypes (i.e., different profiles of circulating and consequently excreted metabolites), likely impacting their effects on health, as it happens for other phenolic metabolites of colonic origin. Well-described examples of these differences are equol production from isoflavones and urolithin production from ellagitannins [30–32]. Stratification of individuals according to their equol/urolithin metabotype has proven to be necessary to understand the health effects associated to isoflavone and ellagitannin intake [33–35]. However, the information on flavan-3-ol colonic metabolites is much less defined. In vitro anaerobic incubations of (−)-epicatechin revealed inter-individual differences in its colonic metabolism and the formation of certain metabolites was correlated with specific microbial phyla [36]. In a recent preliminary study, three putative metabotypes after green tea flavan-3-ol consumption were defined in vivo on the basis of a different urinary production of PVLs and 3-(hydroxyphenyl)propanoic acids (HPPs), through explorative partial least squares-discriminant analysis (PLS-DA) models [26]. Similar results were obtained after consumption of nut proanthocyanidins in nearly free living conditions, using the k-means clustering algorithm [25], but the authors did not associate the different profiles of PVLs and HPPs to metabotypes as they adhered to a more restrictive definition of phenolic metabotypes, characterized by the presence/absence of specific metabolites. However, the urinary profiles there described could be defined as flavan-3-ol colonic metabotypes when considering a broader definition of the term, commonly accepted in the nutrition field as “subgroups of individuals sharing the same metabolic profile” [37]. Beyond terminology, it is clear that there is a lack of information on how to handle the inter-individual variability in the production of phenolic metabolites to define metabotypes in those cases where all the subjects produce all the phenolic metabolites of a catabolic pathway, but in different proportions, as it happens for flavan-3-ols and for the main dietary classes of (poly)phenols.

The primary aim of the present study was to evaluate the existence of metabotypes, based on the urinary excretion of flavan-3-ol metabolites after consumption of flavan-3-ols from cranberry products, to shed light on this key aspect associated with the metabolism of these major phenolics. Secondly, this work aimed at investigating the impact of the statistical techniques used for the definition of phenolic metabotypes, defining an approach to specifically seek for metabotypes when they are not characterized by the dichotomic production/non-production of specific phenolic metabolites.

## Materials and methods

### Intervention studies

The dataset for this study consisted of urinary concentrations of several gut microbiota-derived metabolites of flavan-3-ols, namely monohydroxyPVLs (isomers 3ʹ and 4ʹ), dihydroxyPVLs (3ʹ,4ʹ), and HPPs, quantified in urine samples collected in two different cranberry feeding studies, one with an acute design and one chronic. These metabolites were chosen according to previous evidence [26].

The acute study was a crossover, randomized, controlled intervention trial registered under the NIH ClinicalTrials.gov website (NCT02517775). The study was conducted in accordance with the guidelines stated in the current revision of the Declaration of Helsinki, and informed consent was obtained for all subjects. All procedures involving human subjects were approved by the University of Dusseldorf Research Ethics Committee (ref: 14–012). Briefly, ten healthy men had to consume a cranberry drink containing increasing amounts of total flavan-3-ols (TF) or an isocaloric control (0 mg TF) drink with one-week washout [23, 38]. Participants were instructed to follow a low-(poly)phenol diet for 3 days before and during the study day and had to fast for 12 h before the study day. Urine samples were collected at baseline, between 0–8 h and 8–24 h after drink intake. For the study purpose, data on cumulative urinary excretion (0–24 h) of the metabolites after higher flavan-3-ol intake (716, 1131, 1396, and 1741 mg TF) were considered, for a total of 40 observations. Quantitative data on the urinary excretion of PVLs have been previously reported [23], while data on HPPs are novel.

The chronic study was a parallel, randomized, controlled trial in which 22 healthy participants were asked to consume a cranberry powder containing 0.5 mg of flavan-3-ol monomers and 374 mg of proanthocyanidins every day for one month, without any other dietary restriction or recommendation. The study was registered under the NIH ClinicalTrials.gov website (NCT02764749) and was conducted according to the guidelines laid down in the current revision of the Declaration of Helsinki. Informed consent was obtained for all participants and all procedures involving human subjects were approved by the University of Dusseldorf Research Ethics Committee (Ref: 5360R). Cumulative 24-h urine samples from the first (v1) and the last (v2) intervention day were collected and analyzed to obtain data on metabolite concentration, for a total of 43 observations (1 sample from v1 was missing). In this case, both data on PVLs and HPPs are new. To sum up, a reasonable number of observations (*n* = 83) was used for subsequent statistical analyses.

### Sample analysis

Urine samples were prepared according to a previous report [39] and then analyzed through UHPLC DIONEX Ultimate 3000 fitted with a TSQ Vantage triple quadrupole mass spectrometer, equipped with a heated-electrospray ionization (H-ESI-II) source (Thermo Fisher Scientific Inc., San Jose, CA, USA). Chromatographic and ionization parameters were set following a validated method optimized for the analysis of PVLs [39]. Metabolite identification was carried out by comparison of the retention time with in-house synthesized standards and/or MS/MS fragmentation patterns. Up to 76 compounds among PVLs, PVAs and HPPs were simultaneously monitored in selective reaction monitoring (SRM) mode. Eleven metabolites were quantified in urine samples from the two interventions, namely 5-phenyl-γ-valerolactone-3ʹ-sulfate, 5-phenyl-γ-valerolactone-3ʹ-glucuronide, 5-phenyl-γ-valerolactone-4ʹ-glucuronide, 5-(3ʹ,4ʹ-dihydroxyphenyl)-γ-valerolactone, 5-(hydroxyphenyl)-γ-valerolactone-sulfate (3ʹ,4ʹ isomers), 5-(4ʹ-hydroxyphenyl)-γ-valerolactone-3ʹ-glucuronide, 5-(3ʹ-hydroxyphenyl)-γ-valerolactone-4ʹ-glucuronide, 5-phenyl-γ-valerolactone-methoxy-sulfate isomer (3ʹ,4ʹ), 5-phenyl-γ-valerolactone-sulfate-glucuronide isomer (3ʹ,4ʹ), 3-(phenyl)propanoic acid-sulfate and 3-(phenyl)propanoic acid-glucuronide. This nomenclature follows the current recommendations for (poly)phenol catabolites [40]. Quantification was performed with calibration curves of standards, when available. When not available, metabolites were quantified with the most structurally similar compound, as in the case of 5-phenyl-γ-valerolactone-4ʹ-glucuronide, quantified as its isomer 5-phenyl-γ-valerolactone-3ʹ-glucuronide; 5-(4ʹ-hydroxyphenyl)-γ-valerolactone-3ʹ-glucuronide, 5-(3ʹ-hydroxyphenyl)-γ-valerolactone-4ʹ-glucuronide and 5-phenyl-γ-valerolactone-sulfate-glucuronide isomer (3ʹ,4ʹ), quantified as 5-(5′-hydroxyphenyl)-γ-valerolactone-3′-glucuronide; 5-phenyl-γ-valerolactone-methoxy-sulfate isomer (3ʹ,4ʹ), quantified with 5-(3′-hydroxyphenyl)-γ-valerolactone-4′-sulfate (prepared in-house using reported procedures, [39]); and the 3-(phenyl)propanoic acid-sulfate and –glucuronide, quantified as 3-(4ʹ-hydroxyphenyl)propanoic acid-3ʹ-sulfate and 3-(4ʹ-hydroxyphenyl)propanoic acid-3ʹ-glucuronide (Toronto Research Chemicals, Toronto, Canada), respectively. Sums of metabolites belonging to the same aglycone compound were calculated, namely 5-phenyl-γ-valerolactone-3ʹ-sulfate and 5-phenyl-γ-valerolactone-3ʹ-glucuronide for 5-(3′-hydroxyphenyl)-γ-valerolactone aglycone; 5-phenyl-γ-valerolactone-4ʹ-glucuronide for 5-(4ʹ-hydroxyphenyl)-γ-valerolactone; 5-(3ʹ,4ʹ-dihydroxyphenyl)-γ-valerolactone, 5-(hydroxyphenyl)-γ-valerolactone-sulfate (3ʹ,4ʹ isomers), 5-(4ʹ-hydroxyphenyl)-γ-valerolactone-3ʹ-glucuronide, 5-(3ʹ-hydroxyphenyl)-γ-valerolactone-4ʹ-glucuronide, 5-phenyl-γ-valerolactone-methoxy-sulfate isomer and 5-phenyl-γ-valerolactone-sulfate-glucuronide isomer for 5-(3′,4′-dihydroxyphenyl)-γ-valerolactone; and 3-(phenyl)propanoic acid-sulfate and 3-(phenyl)propanoic acid-glucuronide for 3-(hydroxyphenyl)propanoic acid aglycone. This way, two different datasets, one consisting of 83 observations (samples) and 11 variables corresponding to individual metabolites, and one consisting of 83 observations (samples) and 4 variables corresponding to the sums of metabolites belonging to the same aglycone, were considered. All the metabolite data are expressed as μmol excreted in 24 h.

### Unsupervised analyses

#### Principal component analysis

Principal component analysis (PCA) was performed using SIMCA 16.0.1 software (Sartorius Stedim Data Analytics, Umea, Sweden). Both datasets were subjected to several transformations (no transformation, logarithmic transformation, and power transformation) and four mean centering plus scaling methods: 1) neither centering nor scaling, 2) only centering, 3) centering plus unit variance scaling or autoscaling, and 4) centering with Pareto scaling) of the variables, resulting in a total of 24 PCA models. In particular, logarithmic transformation applied was a 10-based logarithm Log (C1*X + C2) where C1 = 1 and C2 = 0 and the power transformation was (C1*X + C2)^C3^ with C1 = 1, C2 = 0 and C3 = 2 [41]. No scaling was taken into consideration as many variables already presented values close to zero. Centering converted all the data to variations around zero instead of around the mean of the data; unit variance scaling used standard deviation as scaling factor, while Pareto scaling used the square root of standard deviation [42]. All models were presented by default with two principal components (PC). The parameters used to assess the quality of each model and subsequent data interpretability were R^2^(X) and Q^2^, namely the model fit (or explained variation) and the predictive ability, respectively.

#### Cluster analysis: cluster identification and consensus

The two datasets, individual metabolites or the sums of metabolites belonging to the same aglycone compound, were separately submitted to cluster analysis after being centered and unit variance scaled. The cluster analysis was carried out using R version 3.6.1. [43]. The cluster analysis was performed using nine different algorithms, namely hierarchical clustering on principal components (HCPC) [44], hierarchical k-means (H-Kmeans), hierarchical partition around medoids (H-PAM), hierarchical fuzzy (H-fuzzy), partition around medoids (PAM) [45], k-means (Kmeans) [46], fuzzy c-means [47], hierarchical [48] and expectation–maximization (EM) [49]. The number of clusters between two and ten clusters was experimented. To maintain results stability, the process was repeated for five times. Twenty-five internal cluster indexes such as Ball Hall, Banfield Raftery, and C index were applied to measure how compact the clusters were. The optimal number of clusters were selected based on the majority voting scheme. Using the identified optimal number of clusters, we developed nine clustering models using the aforementioned algorithms. Majority voting was used to identify the final cluster assignments (final consensus, FC). In addition, clustering was carried out taking into account the scores of each observation for each principal component (PC) after conducing PCAs with autoscaled data.

### Supervised analysis: partial least square-discriminant analysis

PLS-DA on both datasets was performed using SIMCA 16.0.1 software (Sartorius Stedim Data Analytics, Umea, Sweden). Observations were assigned to classes based on the results of cluster analysis and their PC scores. Variables of both datasets were centered and unit variance scaled (autoscaled). Model validity was assessed by R^2^(X), Q^2^, the random permutation test, and CV-ANOVA within the SIMCA package. The identification of the most relevant metabolites from the whole set of metabolites (variable selection) was performed using the Variable Importance in Projection (VIP) scores, estimating the importance of each variable in the projection used in a PLS model [50]: variables with VIP scores greater than 1 were considered important in the given model.

### Univariate statistics

The urinary excretion of individual metabolites, sums of metabolites belonging to the same aglycone compound, and sums of sulfate or glucuronide metabolites per each cluster defined after applying different clustering methods (FC, EM, Kmeans, and PC score-based) were expressed as mean ± standard deviation. The normality of data distribution was checked through the Kolmogorov–Smirnov test. Data homoscedasticity was tested with Levene’s test. Comparisons between two clusters were performed using independent sample *t* test for normally distributed variables or non-parametric Mann–Whitney *U* test for non-normally distributed variables. Comparisons among three clusters were investigated by one-way ANOVA with post hoc Dunnett’s test (all variables were heteroscedastic) for normally distributed variables or non-parametric Kruskal–Wallis test with post hoc pairwise multiple comparison for non-normally distributed variables. Differences were considered significant at *p* value < 0.05. Boxplots were built using the urinary excretion of sums of metabolites belonging to the same aglycone compound. All these univariate statistical analyses were performed using IBM SPSS Statistics version 26 (IBM, Chicago, IL, USA).

## Results

### Effect of data pre-treatment on resulting PCA models

To evaluate the influence of data pre-treatment on the resulting PCA models and derived biological outcomes, several transformations and scaling methods were applied to the two distinct datasets.

**Fig. 1:**
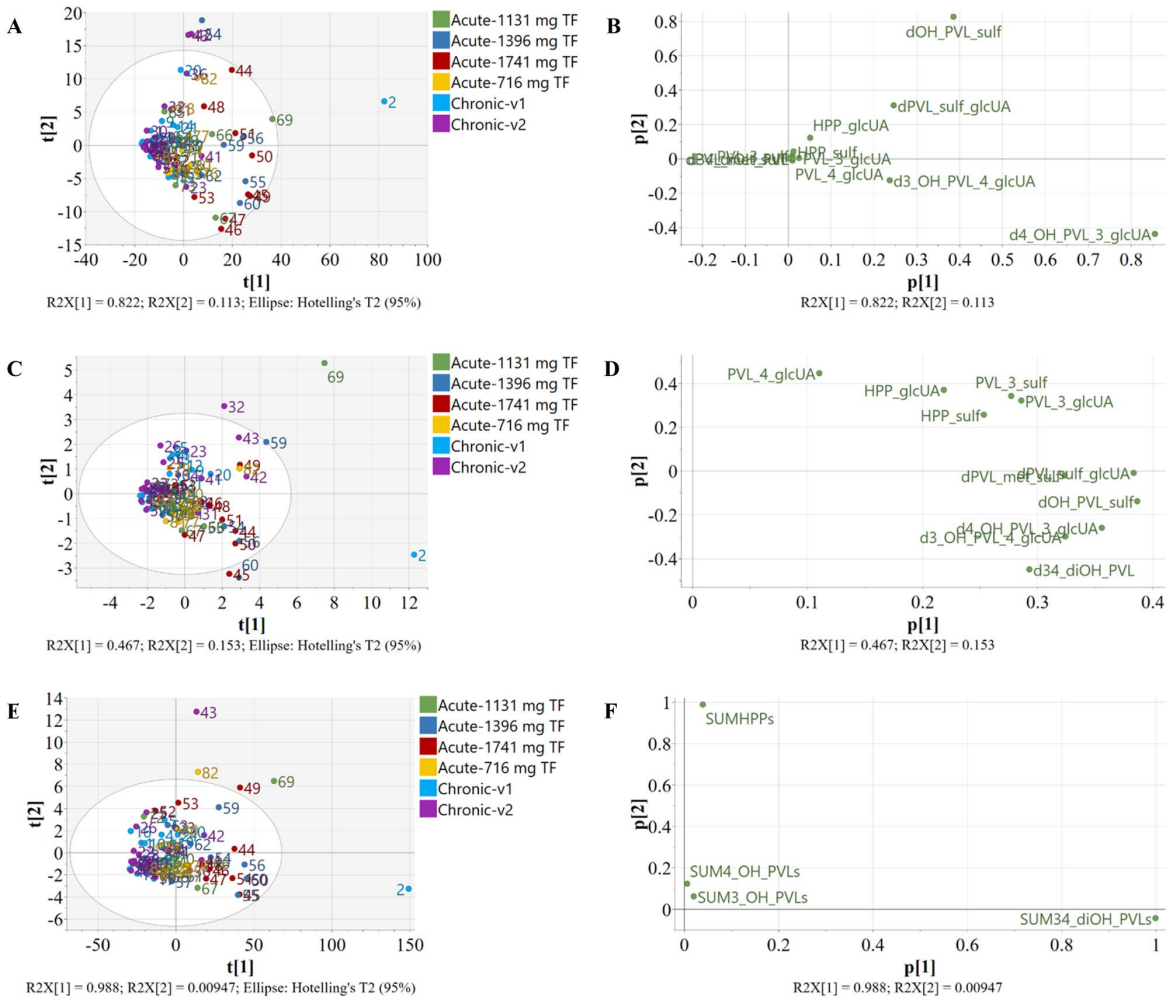
Score (**A, C, E**) and loading (**B, D, F**) plots resulting after PCA analysis on non-transformed, centered data for individual metabolites (**A, B**), non-transformed, centered and unit variance scaled data for individual metabolites (**C, D**), non-transformed, centered data for sums of metabolites belonging to the same aglycone compound (**E, F**)

**Fig. 2:**
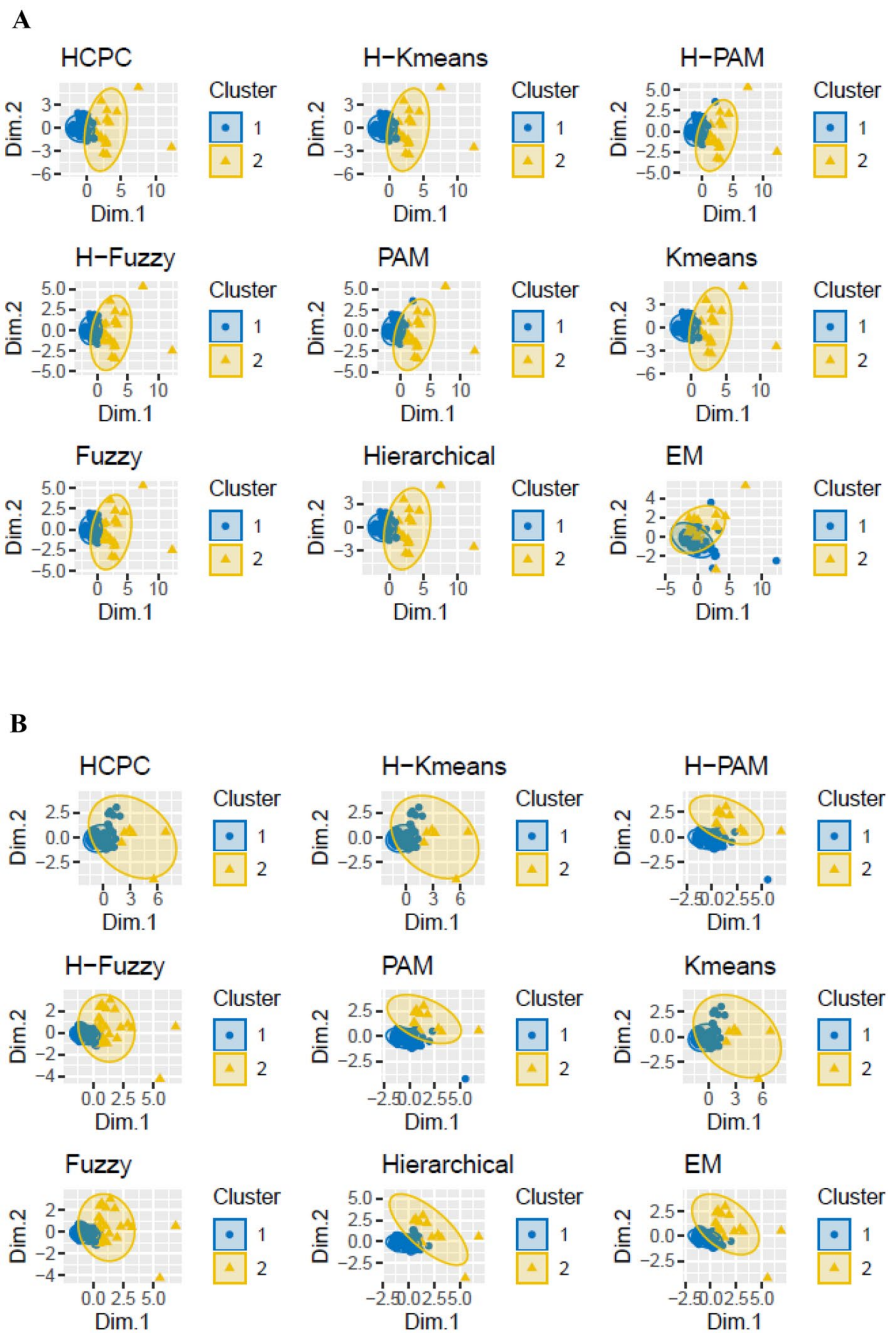
Two-classes cluster plot resulted from the application of different clustering methods on the datasets with individual metabolites (**A**) and with sums of metabolites belonging to the same aglycone compound (**B**)

Considering individual metabolites, applying no data transformation resulted in better models compared to logarithmic and power transformations, which yielded worse R^2^(X) and Q^2^ values than non-transformed models (Table 1). In fact, logarithmic transformation of variables returned many missing values, due to its inability to deal with zero value [42], while power transformation increased data skewness, which is not advisable. This was likely due to the presence of many excretion values close to zero, as it happened for minor excreted metabolites. Regarding scaling methods, for every applied transformation, higher quality models were obtained when using, in order, no scaling > centering > centering with Pareto scaling > centering with unit variance scaling (UV) (or autoscaling) (Table 1). Looking at every model, it was possible to identify patterns of metabolite (variable) distribution in the loading plot (Fig. 1 and Figure S1A-L). Three types of patterns were observed (Table 1): (1) one reflecting differences in phase II metabolism (P), as sulfate and glucuronide metabolites grouped separately in the loading plot (Fig. 1B); (2) one reflecting differences in colonic metabolism (C), on the basis of the derivatives originating from a certain aglycone (Fig. 1D); and (3) one resembling a random distribution (R), as a biological interpretation was not found. A phase II metabolism-based distribution pattern was mainly observed after applying centering or centering with Pareto, while a colonic metabolism-based pattern was shown after autoscaling, regardless of the transformation used (Table 1). This colonic metabolism pattern accounted for the existence of potential flavan-3-ol colonic metabotypes. Interestingly, random distribution was only seen after applying logarithmic transformation. No pattern was associated with the intervention study (acute or chronic) or the treatment/visit type, indicating that these aspects did not influence the variability registered in our data (Fig. 1A and 1C and Figure S1A-L).

**Table 1:** Statistics of computed PCA models illustrating the effect of transformation and scaling methods on the datasets considering individual metabolites and sums of metabolites belonging to the same aglycone compound

Individual metabolites				
Data pre-treatment		Model quality parameters		Pattern*
Transformation	Mean centering + scaling	R^2^X (cum)	Q^2^ (cum)	
None	None	0.965	0.781	C
	Centering	0.935	0.681	P
	Centering + UV	0.621	0.247	C
	Centering + Pareto	0.818	0.551	P
Log	None	0.862	0.622	P
	Centering	0.704	0.303	R
	Centering + UV	0.622	0.230	R
	Centering + Pareto	0.648	0.257	R
Power	None	0.991	0.527	P
	Centering	0.990	0.511	P
	Centering + UV	0.621	-0.070	C
	Centering + Pareto	0.926	0.560	P
Sums of metabolites				
Data pre-treatment		Model quality parameters		Pattern*
Transformation	Mean centering + scaling	R^2^X (cum)	Q^2^ (cum)	
None	None	0.999	0.282	C
	Centering	0.998	0.094	C
	Centering + UV	0.715	− 0.139	C
	Centering + Pareto	0.949	0.076	C
Log	None	0.933	0.396	C
	Centering	0.728	− 0.210	C
	Centering + UV	0.705	− 0.210	C
	Centering + Pareto	0.704	− 0.210	C
Power	None	1.00	0.063	C
	Centering	1.00	0.045	C
	Centering + UV	0.720	− 0.210	C
	Centering + Pareto	0.998	− 0.020	C

The two parameters R^2^X (cum) and Q^2^ (cum) represent, respectively, the model fit (or explained variation) and the predictive ability. The higher these values, the better the model. Abbreviations: UV: Unit Variance. Centering + UV is so-called autoscaling

^*^ “Pattern” stands for “pattern of metabolite distribution”: P, data distribution on the basis of the phase II metabolism; C, distribution on the basis of the colonic metabolism; R, random distribution (no biological explanation)

The best PCA model showing a phase II metabolism-based distribution was obtained after non-transforming and centering data (Fig. 1A and B). Samples in the top right quadrant (Fig. 1A) were characterized by a more abundant excretion of 5-(hydroxyphenyl)-γ-valerolactone-sulfate (3ʹ,4ʹ isomers) (Fig. 1B), while samples in the bottom right quadrant by a higher excretion of glucuronide derivatives of 5-(3ʹ,4ʹ-dihydroxyphenyl)-γ-valerolactone, namely 5-(4ʹ-hydroxyphenyl)-γ-valerolactone-3ʹ-glucuronide and 5-(3ʹ-hydroxyphenyl)-γ-valerolactone-4ʹ-glucuronide. These three compounds are the most excreted after consumption of flavan-3-ols [19, 23, 24] and probably phase II metabolism of some individuals favours sulfation, while in some others glucuronidation is predominant [25, 51]. As a matter of fact, when looking at every subject during the different treatments (acute study) or intervention days (chronic study), it was possible to observe that most of the related samples appeared close together, suggesting that the pattern of conjugation of flavan-3-ol catabolites is preserved in different subjects (as for example samples 44 and 54, 50 and 60, 45 and 55, 47 and 67 in Fig. 1A). On the other hand, the best PCA model representing a colonic metabolism pattern was obtained after non transforming and autoscaling the data (Fig. 1C and D ). In this case, samples in the top right quadrant were characterized by a higher urinary concentration of 3-(hydroxyphenyl)propanoic acid and 5-(hydroxyphenyl)-γ-valerolactone (both 3′ and 4′) derivatives, while samples in the bottom right quadrant were described by a more abundant excretion of 5-(3′,4′-dihydroxyphenyl)-γ-valerolactone derivatives, suggesting differences in the microbial production and urinary excretion of flavan-3-ol catabolites. As in the previous case, samples belonging to the same person fell close and within the same quadrant (as for example samples 49, 59 and 69, 32 and 42, 50 and 60, 44 and 54, 45 and 55, 56 and 66, 47 and 67 in Fig. 1C). This was relevant as it accounted for the conservation of the metabolic pattern in the short time.

When considering sums of metabolites belonging to the same aglycone, the information about phase II metabolism was obviously lost, but it served to better highlight differences in the colonic metabolism of flavan-3-ols. Also, in this case, not transformed data returned higher quality models compared to logarithmic- and power-transformed data (Table 1). Power transformation resulted in overfitted models, especially when coupled to any scaling or centering. Better models were obtained when applying, in order, no scaling > centering > centering with Pareto scaling > centering with unit variance scaling (or autoscaling) to data matrix (Table 1), as for the individual metabolite dataset. All the models showed a colonic metabolism-based distribution pattern, even though not all the models displayed similar distributions for samples and metabolites in the score and loading plots, respectively (Fig. 1E, F and Figure S2A-M). The model resulting after non-transforming and centering the variables in the dataset is shown in Fig. 1E, F, as an example of a high quality PCA model considering sums of metabolites belonging to the same aglycone. The information gathered from these plots was that the samples placed in the top right quadrant were characterized by a higher excretion of 3-(hydroxyphenyl)propanoic acids and those placed in the bottom right quadrant by a higher excretion of 5-(3′,4′-dihydroxyphenyl)-γ-valerolactone derivatives, while 5-(hydroxyphenyl)-γ-valerolactones (both 3′ and 4′ isomers) did not account for sample variability (Fig. 1E, F). In general, the conservative pattern of metabolite production among subjects was also observed using the sum of metabolites (i.e., samples 49, 59 and 69, 46 and 56, 50 and 60, 45 and 55, 44 and 54 in Fig. 1E).

In all these unsupervised analyses, samples out of the Hotelling’s circle (as visible in Fig. 1A, C, and E) were not defined as outliers to be removed, since there are no profiles of metabolite excretion that can be judged as correct and incorrect when it comes to the individual production of flavan-3-ol metabolites.

### Cluster definition

Once PCA highlighted a notable variability in the production of PVLs and HPPs, attention was paid into sample grouping. Testing several clustering criteria on nine clustering algorithms identified two clusters as the optimal number of groups best describing the data. This was done when considering both datasets (individual metabolites, Fig. 2A, and sums of metabolites belonging to the same aglycone compound, Fig. 2B) and all the clustering methods tested performed similarly, except for the EM algorithm when individual data were taken into account (Fig. 2A). Then a FC on clustering was voted, based on each observation frequency to fall within a group (Table S1). The results of three clustering methods were then selected to be used for the PLS-DA, namely EM, since it performed differently to the rest of the clustering algorithms; Kmeans, as it is widely applied [37, 52] and has already been used when studying the potential metabotypization of flavan-3-ol colonic metabolites [25] and FC, because it merged and summarized all the results obtained after testing all the different clustering algorithms. Regarding the distribution of the observations between clusters, one cluster was larger than the other (about 60 observations vs about 20) for all the three algorithms chosen. Of note, a subject allocated in a group after applying a clustering method on the dataset with individual metabolites was not necessarily then allocated in the same group when the same clustering method was applied to the dataset with sums of metabolites belonging to the same aglycone.

Clustering was also carried out according to the scores of each observation for the PCA models obtained after autoscaling. In particular, two (PC score-based, 2 groups) and three clusters (PC score-based, 3 groups) were defined for both datasets. Two clusters were obtained by allocating the observations with a positive PC2 in a group and the observations with a negative PC2 in another group, the two clusters including a similar number of samples. The three clusters were set by allocating the observations with positive PC1 and PC2 scores in one group, the observations with a positive PC1 and a negative PC2 score in a second group, and the observations with a negative PC1 score in a third group. Of these three groups, one was notably more numerous than the other two.

### PLS-DA models to explore differences between clustering methods and the biological relevance of each group

**Fig. 3:**
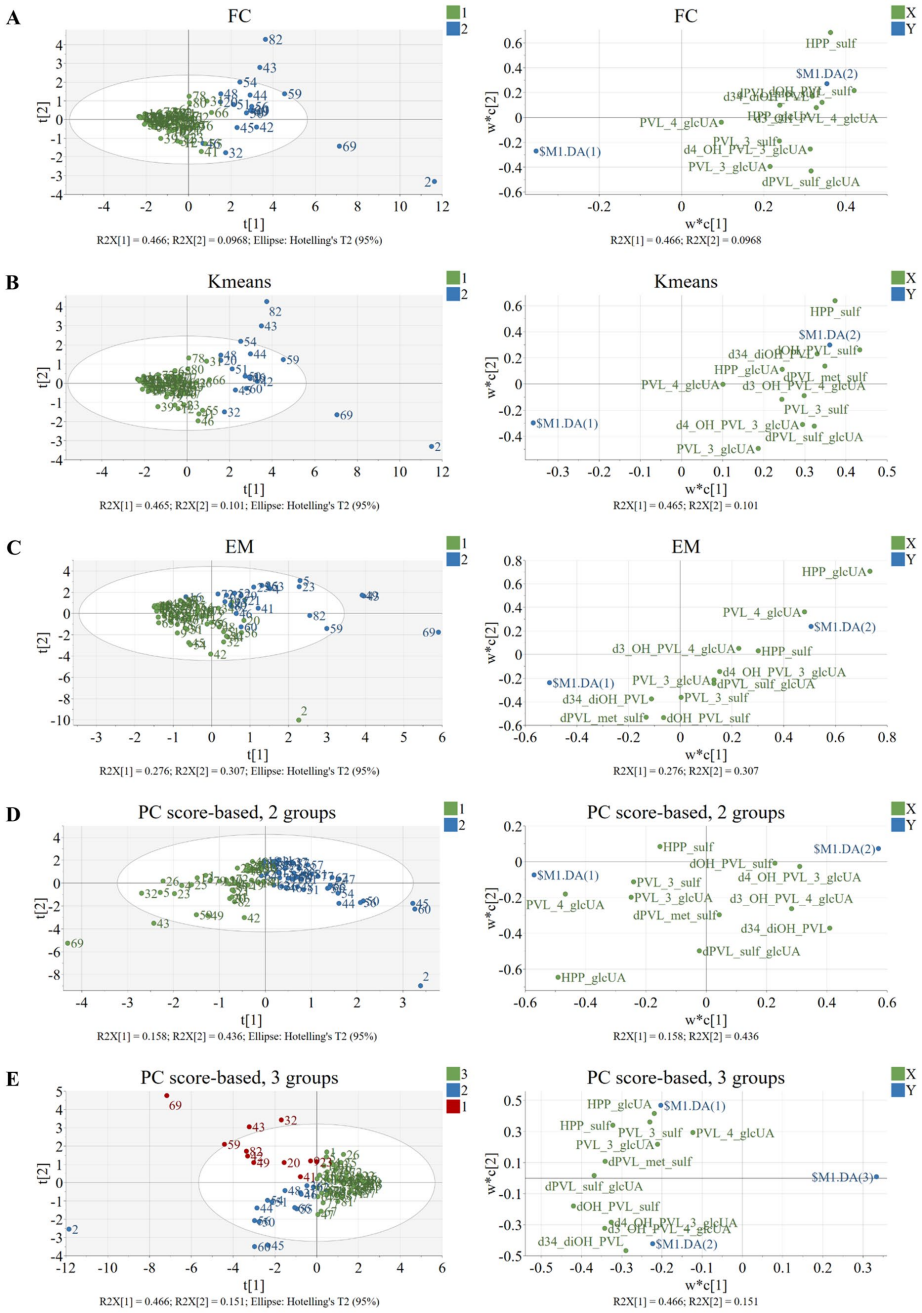
**A-E** PLS-DA models (score and loading plots) considering individual metabolites and the clusters obtained from different clustering methods: (**A**) final consensus –– FC ––, (**B**) k-means –– Kmeans ––, (**C**) expectation–maximization — EM — and PC score-based models for 2 (**D**) or 3 (**E**) groups

All the PLS-DA models performed with individual metabolites taking into account the groups from the selected clustering methods showed a good explained variance (R^2^X) (Table 2). The models performed upon grouping by clustering algorithms (FC, EM, Kmeans) showed a better predictive ability (Q^2^ > 0.5) than the models clustering observations using PC scores (Q^2^ < 0.5). All the models passed cross-validation by CV-ANOVA (*p* value in Table 2) and by random permutation (Figure S3A-E). These results asserted model validation and excluded data overfitting. The two PLS-DA models performed using the classes defined by FC and Kmeans were very similar (Fig. 3A, C). In both models, the distribution of the clusters was due to the amount of metabolites excreted (high vs. low) (VIP values for all the models are reported in Table S2). Differently, the PLS-DA model performed using the classes defined by EM clustering (Fig. 3B) identified two groups characterized by the excretion of different metabolites: group 1 presented a higher excretion of 5-(3′,4′-dihydroxyphenyl)-γ-valerolactone metabolites, while group 2 had a higher excretion of 3-(hydroxyphenyl)propanoic acid derivatives and 5-phenyl-γ-valerolactone-4′-glucuronide, these differences being attributed to differences in the colonic metabolism of flavan-3-ols. A similar trend was observed in the PLS-DA models performed using groups defined according to the PC scores (Fig. 3D and E), as they were described by the excretion of different colonic metabolites. When 2 groups were considered (Fig. 3D) (PC score-based, 2groups), group 1 showed a higher excretion of (monohydroxyphenyl)-γ-valerolactone (both 3′ and 4′) and 3-(hydroxyphenyl)propanoic acid derivatives, while group 2 presented a higher excretion of 5-(3′,4′-dihydroxyphenyl)-γ-valerolactone metabolites. When 3 groups were taken into account (Fig. 3E) (PC score-based, 3groups), group 1 was characterized by a greater excretion of 5-(hydroxyphenyl)-γ-valerolactone (both 3′ and 4′) and 3-(hydroxyphenyl)propanoic acid conjugates, group 2 by a higher excretion of mono-conjugated 5-(3′,4′-dihydroxyphenyl)-γ-valerolactones and group 3 by a limited excretion of metabolites. None of the 5 PLS-DA models showed a distribution of the observations within each group due to phase II metabolism (Fig. 3A-E).

**Table 2:** Statistics of computed PLS-DA models, considering the class resulted from different clustering methods and individual metabolites or sums of metabolites belonging to the same aglycone compound

Individual metabolites				
Clustering method	Model quality		Model reliability	Pattern*
	R^2^X (cum)	Q^2^ (cum)	*p* value CV-ANOVA	
FC	0.562	0.518	4.2e^−12^	A
Kmeans	0.565	0.590	4.6e^−15^	A
EM	0.583	0.538	9.6e^−14^	C
PC score-based, 2 groups	0.594	0.481	2.3e^−10^	C
PC score-based, 3 groups	0.618	0.451	3.8e^−20^	C
Sums of metabolites				
Clustering method	Model quality		Model reliability	Pattern*
	R^2^X (cum)	Q^2^ (cum)	*p* value CV-ANOVA	
FC	0.712	0.601	6.1e^−14^	A
Kmeans	0.635	0.492	3.4e^−10^	A
EM	0.711	0.58	2.5e^−13^	A
PC score-based, 2 groups	0.713	0.402	1.0e^−08^	C
PC score-based, 3 groups	0.713	0.452	4.3e^−11^	C

The two parameters R^2^X (cum) and Q^2^ (cum) represent the model fit (or explained variation) and the predictive ability, respectively. The higher these values, the better the model

Abbreviations: *p* value CV-ANOVA is the *p* value resulting from cross-validation analysis assessing the reliability of the model. The model is valid for *p* value < 0.05

^*^ “Pattern” stands for “pattern of metabolite distribution”: A, data distribution on the basis of the amount of metabolites excreted (high vs. low); C, data distribution on the basis of the colonic metabolism

When considering sums of metabolites belonging to the same aglycone, all the PLS-DA models exhibited good explained variance (R^2^X) (Table 2), even better than what observed for the PLS-DA models with individual metabolites (Table 2). The models performed using classes defined by FC and EM also showed quite good predictive ability (Q^2^ > 0.5), differently from Kmeans and clustering by PC scores (Q^2^ < 0.5). All the models passed cross-validation by CV-ANOVA (*p *value in Table 2) and by random permutation (Figure S4A-E). These results validated the model and excluded overfitting of the data.

The idea behind sums of metabolites is to highlight colonic metabolism by hiding individual differences attributed to phase II metabolism. Nevertheless, PLS-DA models performed using classes defined by clustering algorithms (FC, EM, Kmeans) yielded similar outputs and presented data distributions based mainly on the amount of metabolites excreted (Table 2, Figure S5A-C, VIP values in Table S2). Briefly, most of the samples were characterized by a low excretion of metabolites whereas a smaller group showed a higher excretion of all of them. A distribution into groups reflecting a different colonic metabolism was, however, observed in the PLS-DA models obtained using clusters defined by the PC scores (Figure S5D, E). When 2 groups were defined, one group of observations was characterized by the prevalent excretion of 5-(3′,4′-dihydroxyphenyl)-γ-valerolactones and 5-(3ʹ-hydroxyphenyl)-γ-valerolactones, while the other group was characterized by the excretion of 5-(4ʹ-hydroxyphenyl)-γ-valerolactone and 3-(hydroxyphenyl)propanoic acid derivatives (Figure S5D). When observations were clustered into 3 groups (Figure S5E), one group showed a high excretion of 5-(4ʹ-hydroxyphenyl)-γ-valerolactone and 3-(hydroxyphenyl)propanoic acid derivatives, a second group of 5-(3′,4′-dihydroxyphenyl)-γ-valerolactones and 5-(3ʹ-hydroxyphenyl)-γ-valerolactones, and a third larger group was associated to a scarce excretion of metabolites.

### Univariate statistics confirm the differences in metabolite excretion between groups

Results from PLS-DA models and information on discriminating metabolites were confirmed by univariate statistics. Considering individual metabolites (Table 3), after clustering on the basis of FC and Kmeans, the small group of high excretors of flavan-3-ol catabolites showed statistically significant differences in the urinary excretion of most of the metabolites in comparison with the larger group of low excretors (Table 3), except for 5-phenyl-γ-valerolactone-4′-glucuronide (and 5-phenyl-γ-valerolactone-3′-glucuronide in the case of the Kmeans-based groups). On the contrary, EM clustering identified significant differences only in the quantities of 5-phenyl-γ-valerolactone-4′-glucuronide, 5-(3′-hydroxyphenyl)-γ-valerolactone-4′-glucuronide and 3-(phenyl)propanoic acid-glucuronide, these compounds being excreted at higher levels in the group with a lower number of observations. PC score-based clustering into two groups distinguished a cluster of subjects excreting higher amounts of 5-(hydroxyphenyl)-γ-valerolactones (both 3′ and 4′ derivatives) and 3-(phenyl)propanoic acid-glucuronide and lower quantities of some 5-(3′,4′-dihydroxyphenyl)-γ-valerolactone derivatives, while the other cluster showed an inverse excretion pattern, clearly marked by the differential colonic metabolism of flavan-3-ols (Table 3). When three PC score-based groups were considered, one larger group was characterized by a low excretion of all metabolites (low excretors), another smaller group by a high excretion of 5-(hydroxyphenyl)-γ-valerolactone (both 3′ and 4′ isomers) and 3-(hydroxyphenyl)propanoic acid conjugates, as well as a moderate excretion of main 5-(3′,4′-dihydroxyphenyl)-γ-valerolactone derivatives, and a third small group by a low/moderate excretion of 5-(hydroxyphenyl)-γ-valerolactone (3′/4′) and 3-(hydroxyphenyl)propanoic acid conjugates, and a high excretion of main 5-(3′,4′-dihydroxyphenyl)-γ-valerolactone derivatives (Table 3). In this model, all the metabolites reported statistically significant differences among groups.

**Table 3:** Urinary excretion of individual metabolites and sums of metabolites sulfate or glucuronide per each cluster defined after applying different clustering methods (final consensus—FC—, expectation–maximization—EM—, k-means—Kmeans—, and PC score-based models for 2 or 3 groups) on the dataset with individual metabolites. Data are expressed in µmol (mean ± SD). Mean differences between groups were considered significant for *p* < 0.05 and significant *p* values are highlighted in bold. *N* indicates the number of observations per cluster

Metabolite	Cluster N	FC			Kmeans			EM			PC score-based, 2 groups			PC score-based, 3 groups		
		N	Mean ± St.Dev	*p* value	N	Mean ± St.Dev	*p* value	N	Mean ± St.Dev	*p* value	N	Mean ± St.Dev	*p* value	N	Mean ± St.Dev	*p* value
5-Phenyl-γ-valerolactone-3′-glucuronide	1	65	0.33 ± 0.42	**0.041**	66	0.35 ± 0.47	0.077	60	0.42 ± 0.67	0.261	*40*	*0.68* ± *0.91*	***0.014***	*11*	*1.26* ± *1.39 a*	***0.006***
	2	18	1.06 ± 1.40		17	1.01 ± 1.42		23	0.64 ± 1.06		*43*	*0.31* ± *0.64*		*15*	*0.64* ± *1.00 ab*	
	3	-	-	-	-	-	-	-	-	-	*-*	*-*	*-*	*57*	*0.29* ± *0.42 b*	
5-Phenyl-γ-valerolactone-3′-sulfate	1	*65*	*0.07* ± *0.16*	***0.000***	*66*	*0.07* ± *0.16*	***0.000***	60	0.15 ± 0.40	0.975	*40*	*0.25* ± *0.49*	***0.039***	*11*	*0.67* ± *0.76 a*	***0.000***
	2	*18*	*0.47* ± *0.72*		*17*	*0.49* ± *0.74*		23	0.16 ± 0.38		*43*	*0.07* ± *0.26*		*15*	*0.16* ± *0.42 b*	
	3	*-*	*-*	*-*	*-*	*-*	*-*	-	-	-	*-*	*-*	*-*	*57*	*0.05* ± *0.13 b*	
5-Phenyl-γ-valerolactone-4′-glucuronide	1	65	0.90 ± 0.94	0.118	66	0.90 ± 0.94	0.102	*60*	*0.72* ± *0.58*	***0.002***	*40*	*1.42* ± *1.18*	***0.000***	11	1.84 ± 1.28 a	**0.005**
	2	18	1.30 ± 0.95		17	1.32 ± 0.98		*23*	*1.69* ± *1.33*		*43*	*0.58* ± *0.38*		15	0.86 ± 0.37 b	
	3	-	-	-	-	-	-	*-*	*-*	*-*	*-*	*-*	*-*	57	0.85 ± 0.92 b	
5-(3′,4′-Dihydroxyphenyl)-γ-valerolactone	1	*65*	*0.05* ± *0.10*	***0.000***	*66*	*0.04* ± *0.10*	***0.000***	60	0.12 ± 0.22	0.339	*40*	*0.02* ± *0.08*	***0.000***	*11*	*0.08* ± *0.14 b*	***0.000***
	2	*18*	*0.32* ± *0.32*		*17*	*0.34* ± *0.32*		23	0.07 ± 0.15		*43*	*0.18* ± *0.25*		*15*	*0.39* ± *0.32 a*	
	3	*-*	*-*	*-*	*-*	*-*	*-*	-	-	-	*-*	*-*	*-*	*57*	*0.04* ± *0.08 b*	
5-(4′-Hydroxyphenyl)-γ-valerolactone-3′-glucuronide	1	*65*	*10.8* ± *8.28*	***0.000***	*66*	*11.13* ± *8.64*	***0.000***	60	13.52 ± 13.89	0.191	*40*	*10.66* ± *10.02*	***0.003***	11	18.78 ± 14.09 ab	**0.000**
	2	*18*	*28.85* ± *18.56*		*17*	*28.63* ± *19.11*		23	17.84 ± 11.93		*43*	*18.49* ± *15.15*		15	30.77 ± 17.65 a	
	3	*-*	*-*	*-*	*-*	*-*	*-*	-	-	-	*-*	*-*	*-*	57	9.71 ± 7.33 b	
5-(3′-Hydroxyphenyl)-γ-valerolactone-4′-glucuronide	1	*65*	*2.65* ± *2.04*	***0.000***	*66*	*2.80* ± *2.37*	***0.000***	*60*	*3.40* ± *3.72*	***0.015***	40	2.79 ± 2.35	**0.013**	11	5.14 ± 2.74 a	**0.000**
	2	*18*	*8.73* ± *6.60*		*17*	*8.50* ± *6.73*		*23*	*5.47* ± *5.39*		43	5.07 ± 5.35		15	9.52 ± 6.92 a	
	3	*-*	*-*	*-*	*-*	*-*	*-*	*-*	*-*	*-*	-	-	-	57	2.29 ± 1.61 b	
5-(Hydroxyphenyl)-γ-valerolactone-sulfate (3′,4′ isomers)	1	*65*	*3.11* ± *3.54*	***0.000***	*66*	*3.09* ± *3.51*	***0.000***	60	6.40 ± 8.12	0.574	*40*	*4.41* ± *6.04*	*0.074*	11	11.89 ± 7.03 a	**0.000**
	2	*18*	*16.93* ± *8.87*		*17*	*17.80* ± *8.31*		23	5.34 ± 6.46		*43*	*7.68* ± *8.71*		15	15.80 ± 9.90 a	
	3	*-*	*-*	*-*	*-*	*-*	*-*	-	-	-	*-*	*-*	*-*	57	2.44 ± 2.74 b	
5-Phenyl-γ-valerolactone-sulfate-glucuronide isomer (3′,4′)	1	*65*	*3.19* ± *3.04*	***0.000***	*66*	*3.19* ± *3.02*	***0.000***	60	4.23 ± 4.90	0.260	40	4.71 ± 4.44	0.847	*11*	*9.44* ± *4.51 a*	***0.000***
	2	*18*	*9.71* ± *6.49*		*17*	*10.08* ± *6.49*		23	5.57 ± 4.57		43	4.50 ± 5.20		*15*	*8.72* ± *6.89 a*	
	3	*-*	*-*	*-*	*-*	*-*	*-*	-	-	-	-	-	-	*57*	*2.59* ± *2.31 b*	
5-Phenyl-γ-valerolactone-methoxy-sulfate isomer (3′,4′)	1	*65*	*0.01* ± *0.02*	***0.000***	*66*	*0.01* ± *0.02*	***0.000***	60	0.02 ± 0.03	0.252	40	0.02 ± 0.03	0.715	11	0.05 ± 0.04 a	**0.000**
	2	*18*	*0.05* ± *0.04*		*17*	*0.05* ± *0.04*		23	0.01 ± 0.02		43	0.02 ± 0.03		15	0.04 ± 0.03 a	
	3	*-*	*-*	*-*	*-*	*-*	*-*	-	-	-	-	-	-	57	0.01 ± 0.01 b	
3-(Phenyl)propanoic acid-sulfate	1	*65*	*0.10* ± *0.17*	***0.000***	*66*	*0.10* ± *0.17*	***0.000***	60	0.18 ± 0.27	0.081	40	0.35 ± 0.70	0.208	11	1.08 ± 1.02 a	**0.000**
	2	*18*	*0.92* ± *0.83*		*17*	*0.97* ± *0.82*		23	0.52 ± 0.88		43	0.20 ± 0.28		15	0.42 ± 0.34 a	
	3	*-*	*-*	*-*	*-*	*-*	*-*	-	-	-	-	-	-	57	0.08 ± 0.16 b	
3-(Phenyl)propanoic acid-glucuronide	1	*65*	*1.98* ± *1.75*	***0.001***	*66*	*1.98* ± *1.73*	***0.001***	*60*	*1.46* ± *1.20*	***0.000***	*40*	*3.73* ± *2.96*	***0.000***	11	6.00 ± 3.88 a	**0.000**
	2	*18*	*4.54* ± *3.61*		*17*	*4.68* ± *3.67*		*23*	*5.34* ± *2.81*		*43*	*1.43* ± *1.17*		15	2.33 ± 1.28 b	
	3	*-*	*-*	*-*	*-*	*-*	*-*	*-*	*-*	*-*	*-*	*-*	*-*	57	1.92 ± 1.80 b	
SUM of sulfate conjugates	1	*65*	*3.28* ± *3.65*	***0.000***	*66*	*3.27* ± *3.63*	***0.000***	60	6.76 ± 8.47	0.718	*40*	*5.03* ± *6.90*	*0.113*	11	13.69 ± 7.89 a	**0.000**
	2	*18*	*18.38* ± *9.17*		*17*	*19.32* ± *8.50*		23	6.03 ± 7.55		*43*	*7.97* ± *9.07*		15	16.42 ± 10.34 a	
	3	*-*	*-*	*-*	*-*	*-*	*-*	-	-	-	*-*	*-*	*-*	57	2.58 ± 2.86 b	
SUM of glucuronide conjugates	1	*65*	*16.66* ± *10.7*	***0.000***	*66*	*17.17* ± *11.38*	***0.000***	*60*	*19.51* ± *18.24*	***0.001***	40	19.27 ± 15.2	0.107	11	33.02 ± 20.05 a	**0.000**
	2	*18*	*44.47* ± *24.65*		*17*	*44.15* ± *25.37*		*23*	*30.99* ± *17.51*		43	25.88 ± 21.07		15	44.11 ± 24.35 a	
	3	*-*	*-*	*-*	*-*	*-*	*-*	*-*	*-*	*-*	-	-	-	57	15.06 ± 9.31 b	

**Fig. 4:**
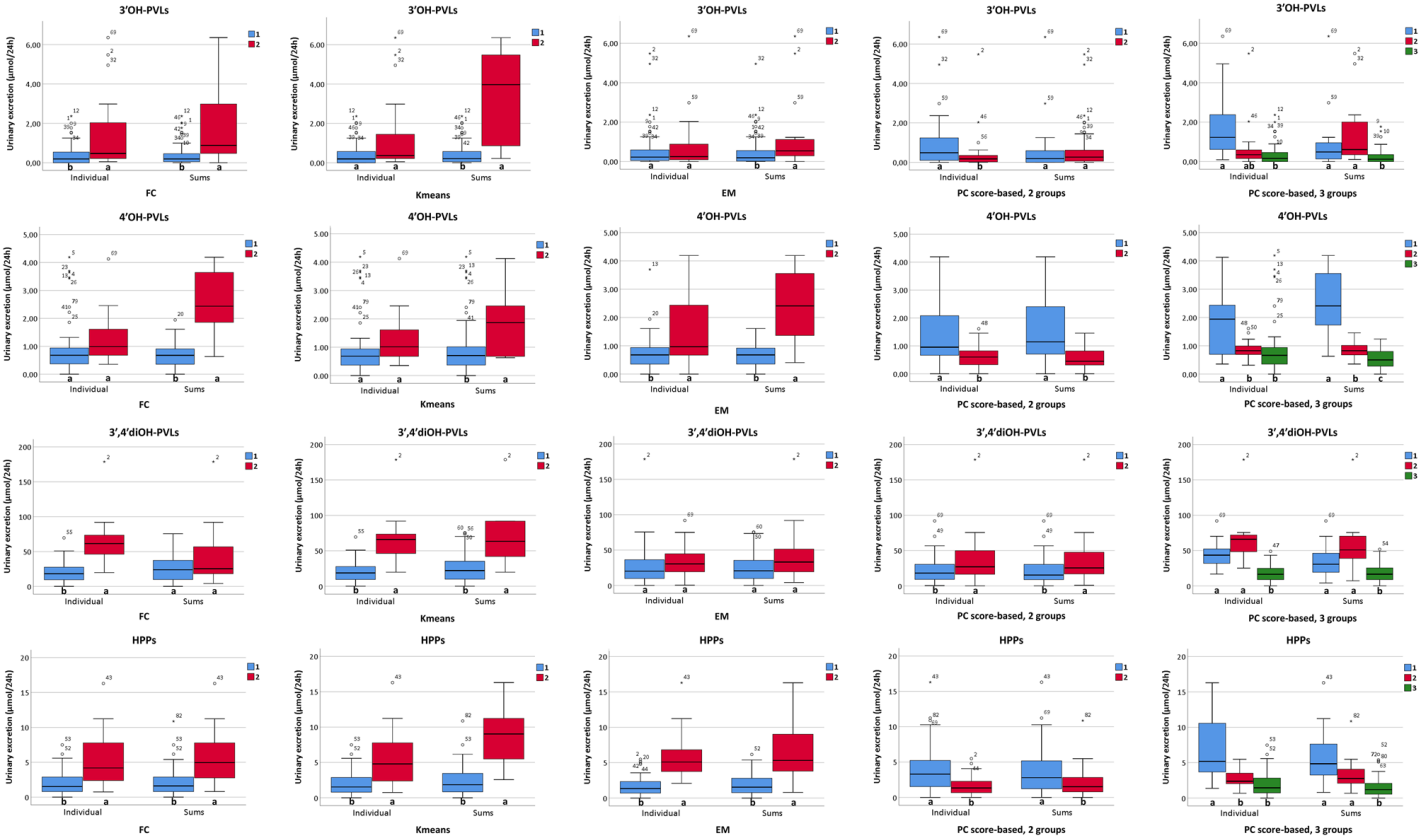
Mean urinary excretion (µmol) over 24 h of sums of metabolites belonging to the same aglycone compound (3ʹOH-PVLs, sum of conjugates from the aglycone 5-(3′-hydroxyphenyl)-γ-valerolactone; 4ʹOH-PVLs, 5-(4ʹ-hydroxyphenyl)-γ-valerolactone; 3ʹ,4ʹdiOH-PVLs, 5-(3′,4′-dihydroxyphenyl)-γ-valerolactone; HPPs, 3-(hydroxyphenyl)propanoic acid), calculated both before and after cluster analysis (“Individual” and “Sums”, respectively). Clustering has been performed on the basis of: Final Consensus (first column), k-means (second column), expectation–maximization algorithm (third column), PC score forming 2 groups (fourth column), PC score forming 3 groups (fifth column). Different letters indicate statistically significant differences (p < 0.05) among groups 1 and 2 or 1, 2 and 3

To favor comparisons between data processing strategies, data for individual metabolites were also pooled, once clusters were defined. Results for sums of metabolites belonging to the same aglycone reflected the same trend previously described for individual metabolites on the basis of each clustering approach (Fig. 4). The general trend described for the dataset with individual metabolites was also confirmed when clustering was performed on the datasets with sums of metabolites belonging to the same aglycone. Nevertheless, some differences in comparison to the previous results were observed. In particular, 5-(3′,4′-dihydroxyphenyl)-γ-valerolactone was not significant between the two clusters for FC model, while differences in 5-(4′-hydroxyphenyl)-γ-valerolactone were statistically significant (Fig. 4). For the Kmeans model, 5-(3′-hydroxyphenyl)-γ-valerolactone and 5-(4′-hydroxyphenyl)-γ-valerolactone were significantly different between the two clusters. 5-(3′-hydroxyphenyl)-γ-valerolactone was significantly different as well in EM model. PC score-based model with 2 groups did not yield statistically significant differences for the sum of 5-(3′-hydroxyphenyl)-γ-valerolactones. Differences in 5-(3′,4′-dihydroxyphenyl)-γ-valerolactone excretion between groups for the PC score-based model with 3 groups were the same as reported when individual data was considered, while for the other aglycones some differences in their excretion were found (Fig. 4).

The sum of metabolites was also conducted by considering phase II metabolism (sulfation and glucuronidation) using the dataset for individual metabolites. After clustering by FC and Kmeans, the small observation groups presented higher excretion of sulfate and glucuronide derivatives (Table 3). Something similar was seen for the PC score-based model on 3 groups, with 2 groups having a higher excretion of phase II conjugates than the third group, but without differences between the 2 groups with a high excretion of PVLs and HPPs. The PC score-based model on 2 groups did not yield differences in the rate of conjugations between groups, while EM clustering returned a group characterized by the higher excretion of glucuronide (Table 3).

### Sulfate/glucuronide ratio as proxy of individual variability in phase II metabolism

**Fig. 5:**
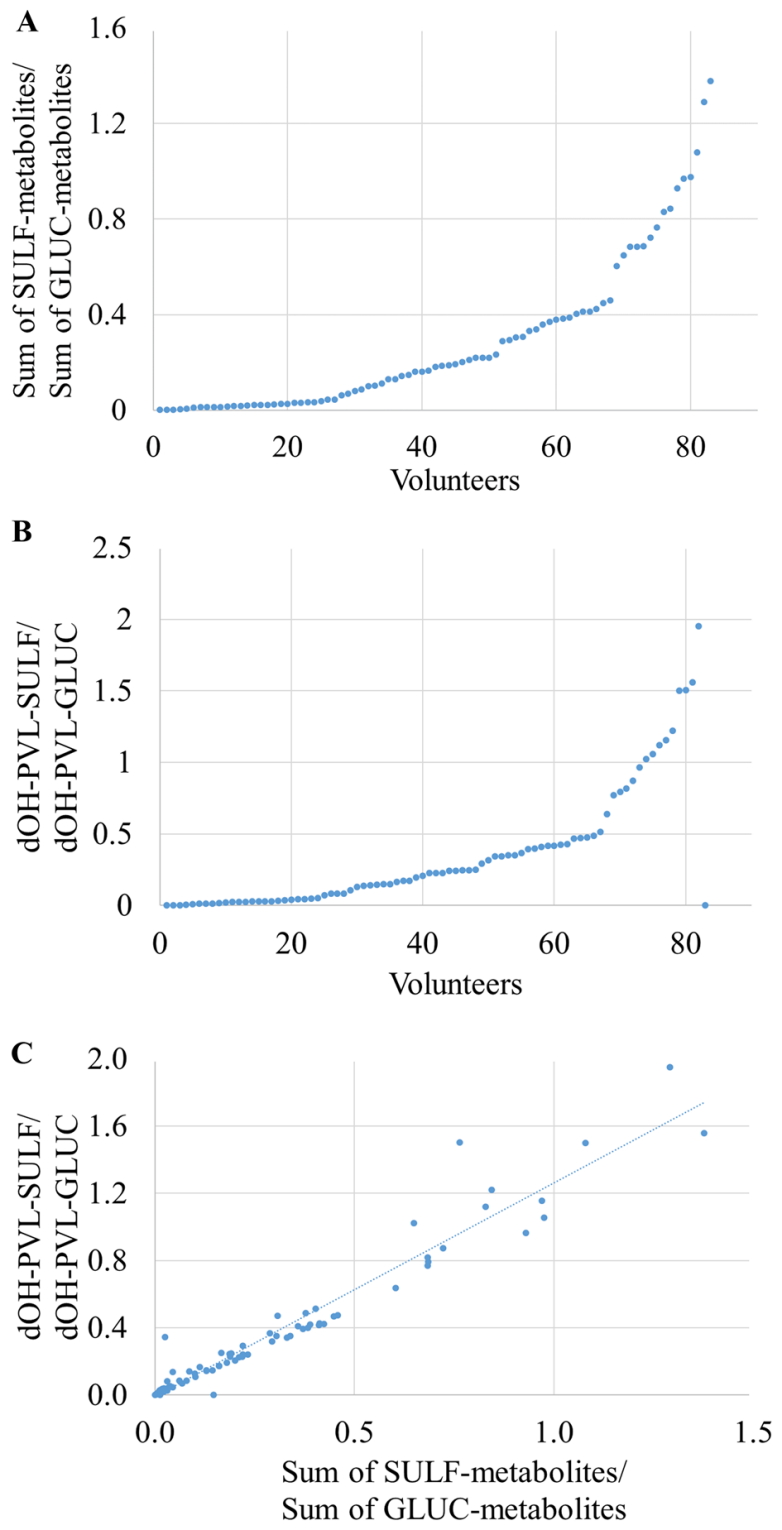
Inter-individual variability in phase II metabolism illustrated by the sulfate (SULF)/glucuronide (GLUC) ratio of the sums of respective conjugated metabolites (**A**) and of 5-(3′,4′-dihydroxyphenyl)-γ-valerolactones (dOH-PVL) (**B**) in urine samples. (**C**) Relationship between the sulfate/glucuronide ratio of all the metabolites and the sulfate/glucuronide ratio of 5-(3′,4′-dihydroxyphenyl)-γ-valerolactones

To investigate deeper the inter-individual variability in phase II metabolism, an aspect that was previously described [25, 51], the sulfate/glucuronide ratios of the sums of all metabolites (Fig. 5A), 5-(3′,4′-dihydroxyphenyl)-γ-valerolactones (Fig. 5B), 5-(hydroxyphenyl)-γ-valerolactones (Figure S6A) and 3-(hydroxyphenyl)propanoic acids (Figure S6B) were calculated. 5-phenyl-γ-valerolactone-sulfate-glucuronide isomer (3ʹ,4ʹ) was excluded from the calculation. In general, the sulfate/glucuronide ratio of the sums of all metabolites (Fig. 5A) and of the sums of aglycones (Fig. 5B, Figure S6A and B) was in favor of glucuronide conjugates. Just very few subjects (*n* = 2 for the sum of all metabolites, *n* = 7 for 5-(3′,4′-dihydroxyphenyl)-γ-valerolactones) presented a ratio higher than 1.1, meaning higher excretion of sulfate conjugates. This observation was in line with one of the PCA models characterized by a more abundant excretion of 5-(hydroxyphenyl)-γ-valerolactone-sulfate (3ʹ,4ʹ isomers, Fig. 1A,B).

A positive linear correlation was clearly observed (r = 0.977, p < 0.001) between the sulfate/glucuronide ratio of the sums of all the metabolites and the sulfate/glucuronide ratio of 5-(3′,4′-dihydroxyphenyl)-γ-valerolactones (Fig. 5C), suggesting that the ratio of the sums of all the sulfate or glucuronide metabolites is mainly influenced by the sulfate/glucuronide ratio of 5-(3′,4′-dihydroxyphenyl)-γ-valerolactones, which were actually the main excreted metabolites. Weaker correlations were observed between the sulfate/glucuronide ratio of the sums of metabolites and the sulfate/glucuronide ratio of both 5-(hydroxyphenyl)-γ-valerolactones (r = 0.547, *p* < 0.001, Figure S6C) or 3-(hydroxyphenyl)propanoic acids (r = 0.724, *p* < 0.001, Figure S6D).

## Discussion

When flavan-3-ols are consumed, different patterns of production and excretion of their main colonic metabolites, namely PVLs and HPPs, can be observed due to the unique genetic asset and microbiota composition of each individual [20–23, 25–29]. For instance, some subjects may be more efficient in metabolizing flavan-3-ols and producing higher quantities of metabolites compared to others [25, 53], or glucuronidation may be favored in some individuals rather than sulfation [25, 51]. Metabotyping may be a strategy to manage this individual variability and to further investigate its consequences in the impact on the observed health effects attributed to flavan-3-ols. This work demonstrates that, by applying different strategies of multivariate data analysis, it is possible to cluster all these different metabolic patterns, characterizing groups of individuals. This outcome confirmed thus the existence of metabotypes in the urinary excretion of flavan-3-ol colonic metabolites, as previously hypothesized [26], even though results were different from those preliminary ones.

Data pre-treatment deeply influenced the observations gathered from PCA. No transformation of the data returned higher quality models. Mean centering and mean centering + Pareto scaling highlighted different patterns of phase II metabolism (sulfation vs. glucuronidation), while centering + UV scaling showed different patterns of colonic metabolism. These facts emphasized the importance of data pre-treatment when analyzing datasets of flavan-3-ol metabolites, as it is well acknowledged that pre-treatment procedures in metabolomics studies may greatly influence the biological relevance of the results [42].

The inter-individual variability in phase II metabolism observed using certain PCA models revealed that most of the subjects excreted higher quantities of glucuronide derivatives, and this was supported by the analysis of the sulfate/glucuronide ratio and, especially, of the sulfate/glucuronide ratio of 5-(3′,4′-dihydroxyphenyl)-γ-valerolactones, quantitatively the main excreted metabolites, as previously discussed [23]. This variability could be related to genetic polymorphisms in phase II enzymes [54–56], but also to the influence of the dose of flavan-3-ols, since the sulfonation pathway has higher affinity but lower capacity than the glucuronidation one, so that when the consumed amount of flavan-3-ols increases, a shift from sulfation toward glucuronidation might occur [57]. This may be an explanation with respect to other works reporting a higher excretion of sulfate derivatives [22, 51], together with the lack of the respective reference compound for metabolite quantification [58], but further research is needed to better understand the reasons behind these differences in phase II metabolism. Therefore, to overcome experimental limitations associated with the production and quantification of phase II metabolites, the sums of metabolites belonging to the same aglycone were taken into account, also as a strategy that should lead to a better assessment of colonic metabolism. However, this reductive approach did not yield any benefits in comparison with processing individual metabolite data and calculating sums at the end of the procedure, in line with a previous report [26]. It is worth mentioning that different PCA data pre-treatments should be assessed to fully understand and summarize what happens at colonic level, as well as in phase II conjugation.

Regarding clustering algorithms, EM, rather than the broadly used Kmeans algorithm, was useful in clustering individuals on the basis of their pattern of excretion of colonic metabolites (i.e., flavan-3-ol colonic metabotypes). Kmeans served its purpose to identify groups of individuals with different metabolic profiles in the production of flavan-3-ol colonic metabolites [25], but, in the present work, it was quite influenced by the overall amount of metabolites excreted. The PLS-DA model built using EM clustering was also affected by the excreted amount of metabolites, but it showed a trend towards a different metabolic profiles, as a group of individuals was characterized by a relatively high excretion of 5-(4′-hydroxyphenyl)-γ-valerolactone and HPP derivatives, while the other by a reduced production of these metabolites. The models better highlighting urinary metabotypes of flavan-3-ol colonic metabolites were the two models built using PC scores for clustering. The model with two groups of observations suggested that a small group of subjects is more able to metabolize flavan-3-ols into smaller metabolites (5-(hydroxyphenyl)-γ-valerolactones—both 3ʹ and 4ʹ- and HPPs), while 5-(3ʹ,4ʹ-dihydroxyphenyl)-γ-valerolactones was predominant in the main group, fully in line with previous data [25, 26]. The model with three groups was able to discriminate observations on the basis of both the total amount excreted and the pattern of colonic metabolites, leading to metabotypes deserving to be further investigated in future bioactivity and functional studies. It should be noted that the PC score-based strategy had the power of well describing the data, but a limited predictive ability. Nevertheless, generalizable predictive models in flavan-3-ol colonic catabolism are not expected due to the chemical complexity of this family of polyphenols and to their variability in dietary sources administered. For example, flavan-3-ols from green tea are rich in trihydroxylated precursors and may lead to more complex metabolic pathways [26], flavan-3-ols from cranberries, the case of the present work, are poor of trihydroxylated precursors and rich in dihydroxylated ones, while the intervention by Cortés-Martín and colleagues [25] consisted of a supplementation of 54.5 mg/d of nut procyanidins, but in free-diet conditions, so that the presence of both dihydroxylated and trihydroxylated precursors is foreseeable according to epidemiological data on the consumption of flavan-3-ols in similar populations [4, 5, 59, 60].

Fecal fermentation of (−)-epicatechin has also highlighted the possible existence of metabotypes among 24 individuals using PCA and hierarchical clustering, where different patterns of (−)-epicatechin catabolism were observed [36]. Common features on metabolic phenotypes have been observed, regardless of the experimental setting, like, for example, the ability of some individuals to metabolise 5-(3ʹ,4ʹ-dihydroxyphenyl)-γ-valerolactone into 5-(hydroxyphenyl)-γ-valerolactones and HPPs at a faster pace and the presence of low producers of all metabolites. However, the allocation of most of the individuals into specific metabotypes will depend on the dataset used, unless further insights on key discriminant metabolites arise, or massive epidemiological evidence is collected to establish specific thresholds.

The biological causes behind the observed metabotypes may rely on the differences in gut microbiota composition of individuals, as this has been reported to be the most important factor modulating the inter-individual variability reported in the colonic metabolism of phenolic compounds [61] and, in particular, of flavan-3-ols [19, 36]. Information on specific bacterial strains and enzymes involved in the bioconversion of flavan-3-ols to PVLs and low molecular weight phenolic acids, as well as on factors that may modulate their activities, is very limited. Up to now, *Adlercreutzia equolifaciens, Eggerthella lenta*, *Flavonifractor plautii*, and *Lactobacillus plantarum* IFPL935 are the only bacteria identified as responsible for the catabolism of flavan-3-ols into 5-(3′,4ʹ-dihydroxyphenyl)-γ-valerolactone and 5-(3ʹ-hydroxyphenyl)-γ-valerolactone [18, 62, 63], but, for example, 5-(4ʹ-hydroxyphenyl)-γ-valerolactone has not been described as one of their catabolic products. In addition, no microorganisms responsible for further β-oxidation into 3-(hydroxyphenyl)propanoic acids have been reported, yet.

Besides a better understanding of the metabolism and bioavailability of flavan-3-ols, the importance of identifying different metabotypes relies on the possibility of unravelling the health effects associated with flavan-3-ol consumption and associated to their microbiota-derived metabolites, as it has been described for isoflavones (with equol production) and ellagitannins (with urolithin production) [33–35]. For instance, in vitro findings support the biological effects of flavan‐3‐ol colonic metabolites against uropathogenic *Escherichia coli* adherence to uroepithelial cells [12, 13], while it is well known that human studies administering cranberry flavan-3-ols to prevent urinary tract infections (UTIs) have reported conflicting results [64, 65]. This might be due to different profiles of excreted metabolites, exerting different biological effects. In this sense, clustering subjects according to their urinary metabotype of flavan-3-ol colonic metabolites may provide new insights in the actual effect of flavan-3-ols on UTI prevention, not only through cranberries but potentially also from other flavan-3-ol food sources like cocoa, wine, pome fruits, other berries, and nuts.

## Conclusion

The current work shed light on the existence of metabotypes in the urinary excretion of flavan-3-ol metabolites, which are not characterized by the production/non-production of specific metabolites, but by different quali-quantitative metabolic profiles. A series of univariate and multivariate tools, all broadly accessible to the research community, highlighted the importance of data pre-treatment and clustering methods on the final outcomes for a given dataset. Different profiles in the urinary excretion of PLVs and HPPs were observed upon cranberry consumption in two diverse experimental settings, these metabolic profiles being related to not only specific pathways of phase II metabolism but also the type of metabolites produced at colonic level. Insights depended on PCA data pre-treatment: non-transformed, centered, and UV-scaled data were key to unravel metabolic patterns based on colonic metabolism, while other approaches favored differences in phase II metabolism. Regarding clustering, while Kmeans and a FC algorithm highlighted differences in the overall production of PVLs and HPPs, the EM algorithm and PC score-based clustering yielded well-defined metabotypes in the urinary excretion of these metabolites. The true physiological relevance of each metabotyping model, whether based on phase II or colonic metabolism, will be related to the application of these inter-individual differences to explore their potential impact on the biological activity of this major (poly)phenol subclass. When applied to physiological outcomes, different ways of metabotyping may lead to different biological observations, fostering the understanding of the impact of flavan-3-ols on human health. The unambiguous elucidation of metabotypes and the allocation of subjects into a metabotype or another, when dealing with (poly)phenols not characterized by the selective production of specific metabolites, will likely depend on the datasets considered for the development of further predictive models. Therefore, these results, both the proposed metabotypes and the defined procedures, should be validated in larger datasets involving a higher number of participants, more phenolic metabolites from the flavan-3-ol metabolic pathway, and different sources of flavan-3-ols. In any case, this work represents an additional step toward the understanding of the bioavailability of flavan-3-ols and the inter-individual variability associated to these compounds and it will be useful for future studies aiming to investigate metabolic phenotypes in the production and urinary excretion of other classes of phenolic metabolites.

## Supplementary Information

Below is the link to the electronic supplementary material.

Supplementary file1 (PDF 4388 KB)

Supplementary file2 (XLSX 18 KB)
