# Supplementary material for: Metabotypes of flavan-3-ol colonic metabolites after cranberry intake: elucidation and statistical approaches
Source: Eur J Nutr. 2021 Nov 9;61(3):1299–317. doi: 10.1007/s00394-021-02692-z (PMC8921115; doi:10.1007/s00394-021-02692-z)
Supplement: Supplementary file 1 — Supplementary file1 (PDF 4388 KB) [file 394_2021_2692_MOESM1_ESM.pdf]

Metabotypes of flavan-3-ol colonic metabolites after cranberry intake: elucidation and statistical approaches

Pedro Mena[1,,+], Claudia Favari[1,+], Animesh Acharjee[2,3,4], Saisakul Chernbumroong[2,3], Letizia Bresciani[1], Claudio Curti[5], Furio Brighenti[1], Christian Heiss[6], Ana Rodriguez-Mateos[7], Daniele Del Rio[1, 8]*

[1] Human Nutrition Unit, Department of Food & Drug, University of Parma, Parma, Italy

[2] College of Medical and Dental Sciences, Institute of Cancer and Genomic Sciences, Centre for Computational Biology, University of Birmingham, Birmingham, B15 2TT, UK

[3] Institute of Translational Medicine, University Hospitals Birmingham NHS, Foundation Trust, Birmingham, B15 2TT, UK

[4] NIHR Surgical Reconstruction and Microbiology Research Centre, University Hospital Birmingham, Birmingham B15 2WB, UK

[5] Department of Food & Drug, University of Parma, Parma, Italy

[6] Department of Clinical and Experimental Medicine, Faculty of Health and Medical Sciences, University of Surrey, Guildford, UK

[7] Department of Nutritional Sciences, Faculty of Life Sciences and Medicine, King's College London, London, UK

[8] School of Advanced Studies on Food and Nutrition, University of Parma, Parma, Italy

[+] Equal contributors

[*] Corresponding author: Via Volturno 39, 43125 Parma, Italy; Tel.: +39 0521-903841; E-mail: pedro.mena@unipr.it

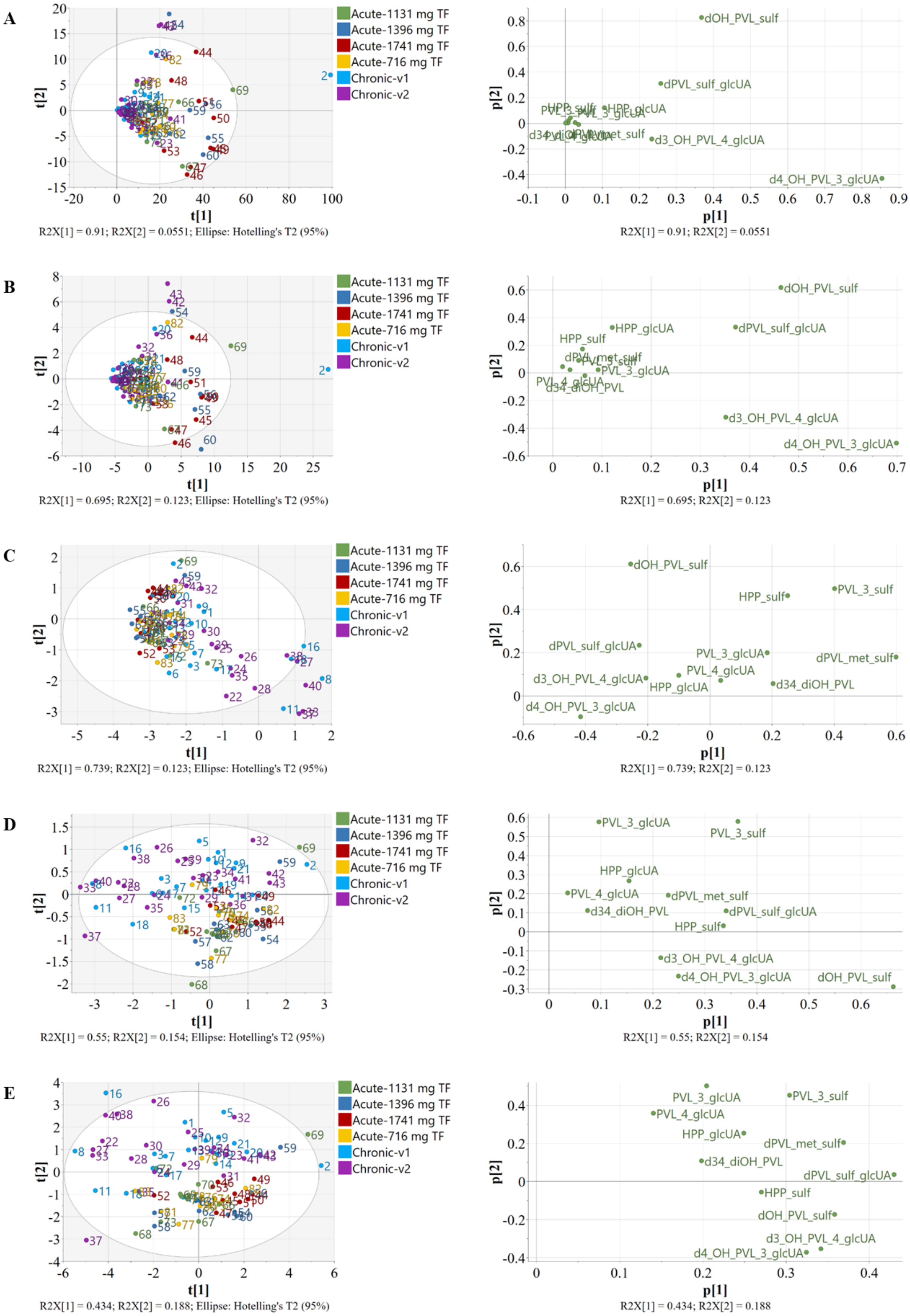
A
t[2]
t[1]
Acute-1131 mg TF
Acute-1396 mg TF
Acute-1741 mg TF
Acute-716 mg TF
Chronic-v1
Chronic-v2
R2X[1] = 0.91; R2X[2] = 0.0551; Ellipse: Hotelling's T2 (95%)
p[2]
p[1]
dOH_PVL_sulf
dPVL_sulf_glcUA
d3_OH_PVL_4_glcUA
d4_OH_PVL_3_glcUA
R2X[1] = 0.91; R2X[2] = 0.0551
B
t[2]
t[1]
Acute-1131 mg TF
Acute-1396 mg TF
Acute-1741 mg TF
Acute-716 mg TF
Chronic-v1
Chronic-v2
R2X[1] = 0.695; R2X[2] = 0.123; Ellipse: Hotelling's T2 (95%)
p[2]
p[1]
dOH_PVL_sulf
HPP_glcUA
dPVL_sulf_glcUA
HPP_sulf
PVL_3_glcUA
d3_OH_PVL_4_glcUA
d4_OH_PVL_3_glcUA
R2X[1] = 0.695; R2X[2] = 0.123
C
t[2]
t[1]
Acute-1131 mg TF
Acute-1396 mg TF
Acute-1741 mg TF
Acute-716 mg TF
Chronic-v1
Chronic-v2
R2X[1] = 0.739; R2X[2] = 0.123; Ellipse: Hotelling's T2 (95%)
p[2]
p[1]
dOH_PVL_sulf
HPP_sulf
PVL_3_sulf
dPVL_sulf_glcUA
PVL_3_glcUA
dPVL_met_sulf
PVL_4_glcUA
d3_OH_PVL_4_glcUA
HPP_glcUA
d34_diOH_PVL
d4_OH_PVL_3_glcUA
R2X[1] = 0.739; R2X[2] = 0.123
D
t[2]
t[1]
Acute-1131 mg TF
Acute-1396 mg TF
Acute-1741 mg TF
Acute-716 mg TF
Chronic-v1
Chronic-v2
R2X[1] = 0.55; R2X[2] = 0.154; Ellipse: Hotelling's T2 (95%)
p[2]
p[1]
PVL_3_glcUA
PVL_3_sulf
HPP_glcUA
PVL_4_glcUA
dPVL_met_sulf
d34_diOH_PVL
dPVL_sulf_glcUA
HPP_sulf
d3_OH_PVL_4_glcUA
d4_OH_PVL_3_glcUA
dOH_PVL_sulf
R2X[1] = 0.55; R2X[2] = 0.154
E
t[2]
t[1]
Acute-1131 mg TF
Acute-1396 mg TF
Acute-1741 mg TF
Acute-716 mg TF
Chronic-v1
Chronic-v2
R2X[1] = 0.434; R2X[2] = 0.188; Ellipse: Hotelling's T2 (95%)
p[2]
p[1]
PVL_3_glcUA
PVL_3_sulf
PVL_4_glcUA
HPP_glcUA
dPVL_met_sulf
d34_diOH_PVL
dPVL_sulf_glcUA
HPP_sulf
dOH_PVL_sulf
d3_OH_PVL_4_glcUA
d4_OH_PVL_3_glcUA
R2X[1] = 0.434; R2X[2] = 0.188

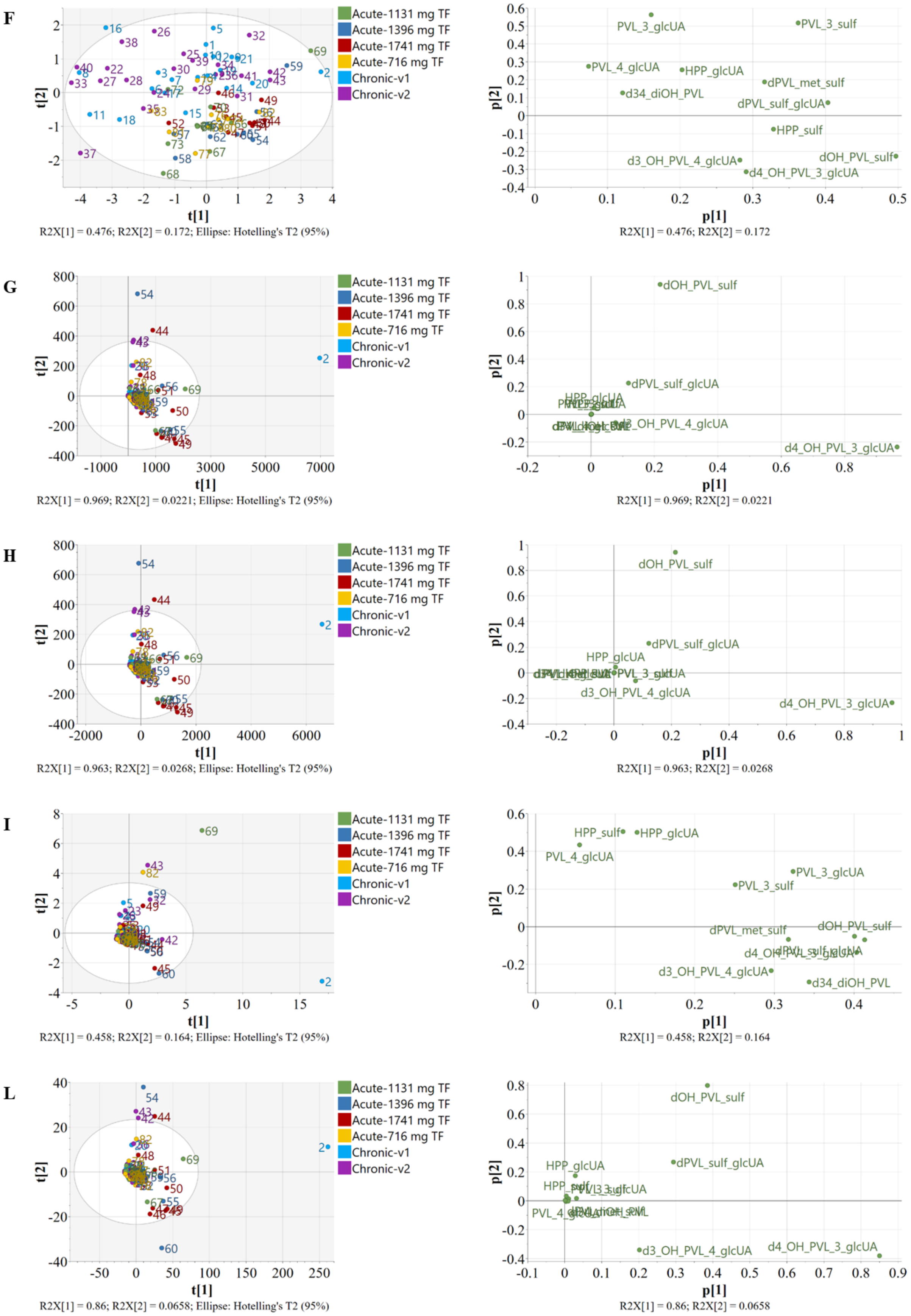

Figure S1A-L. PCA models (score and loading plots) for individual metabolites on: (**A**) non-transformed non-scaled data, (**B**) non-transformed centered and Pareto scaled data, (**C**) log-

transformed non-scaled data, (**D**) log-transformed centered data, (**E**) log-transformed autoscaled data, (**F**) log-transformed centered and Pareto scaled data, (**G**) power-transformed non-scaled data, (**H**) power-transformed centered data, (**I**) power-transformed autoscaled data, (**L**) power-transformed centered and Pareto scaled data.

A

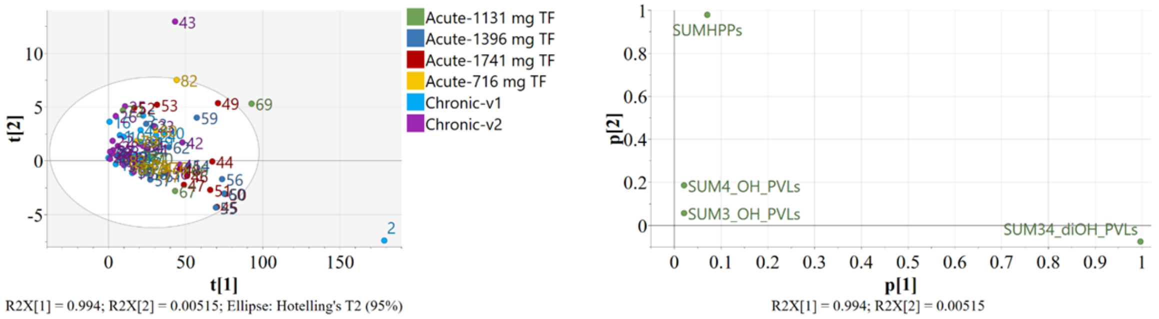
Acute-1131 mg TF
Acute-1396 mg TF
Acute-1741 mg TF
Acute-716 mg TF
Chronic-v1
Chronic-v2
t[2]
t[1]
R2X[1] = 0.994; R2X[2] = 0.00515; Ellipse: Hotelling's T2 (95%)
SUMHPPs
SUM4_OH_PVLs
SUM3_OH_PVLs
SUM34_diOH_PVLs
p[2]
p[1]
R2X[1] = 0.994; R2X[2] = 0.00515

B

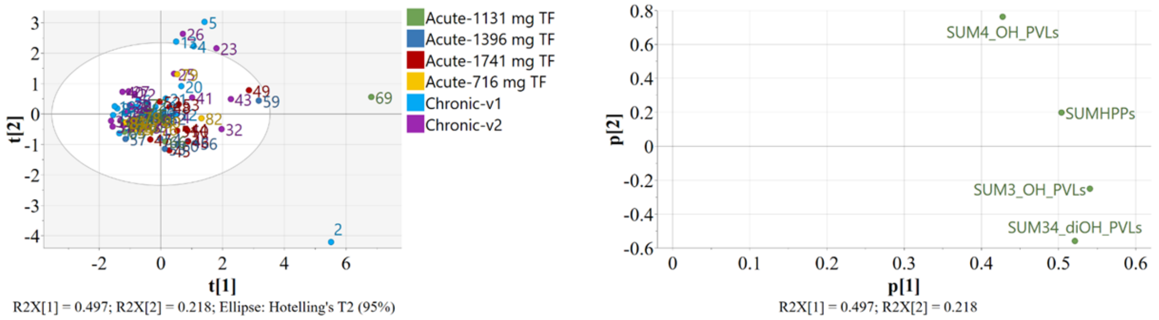
Acute-1131 mg TF
Acute-1396 mg TF
Acute-1741 mg TF
Acute-716 mg TF
Chronic-v1
Chronic-v2
t[2]
t[1]
R2X[1] = 0.497; R2X[2] = 0.218; Ellipse: Hotelling's T2 (95%)
SUM4_OH_PVLs
SUMHPPs
SUM3_OH_PVLs
SUM34_diOH_PVLs
p[2]
p[1]
R2X[1] = 0.497; R2X[2] = 0.218

C

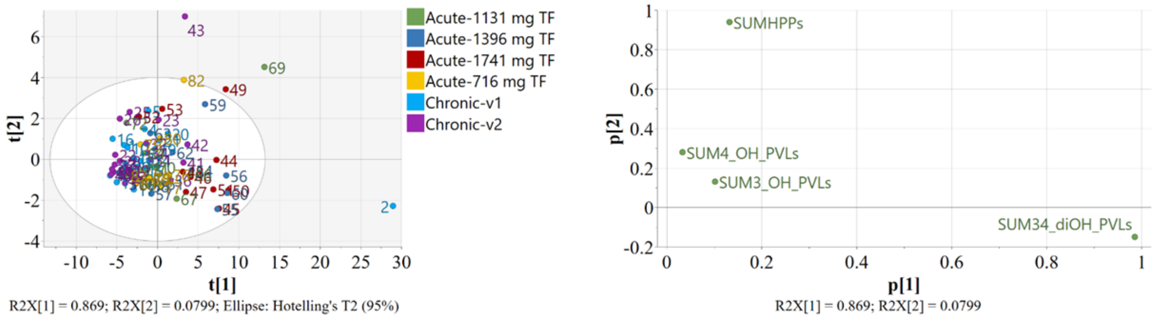
Acute-1131 mg TF
Acute-1396 mg TF
Acute-1741 mg TF
Acute-716 mg TF
Chronic-v1
Chronic-v2
t[2]
t[1]
R2X[1] = 0.869; R2X[2] = 0.0799; Ellipse: Hotelling's T2 (95%)
SUMHPPs
SUM4_OH_PVLs
SUM3_OH_PVLs
SUM34_diOH_PVLs
p[2]
p[1]
R2X[1] = 0.869; R2X[2] = 0.0799

D

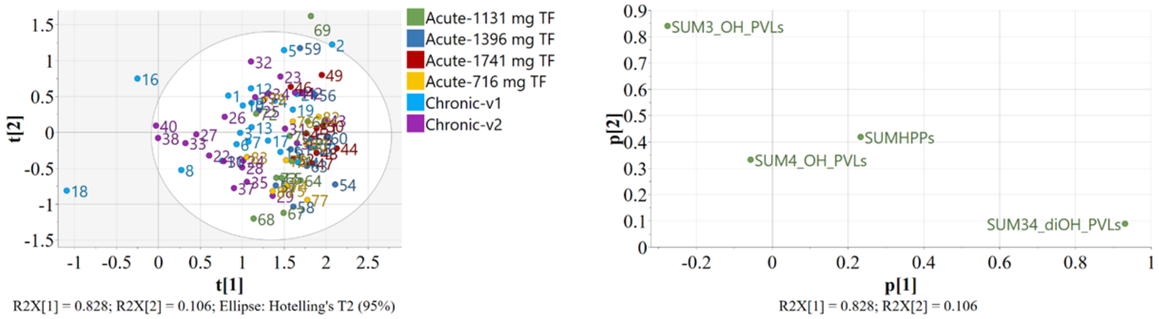
Acute-1131 mg TF
Acute-1396 mg TF
Acute-1741 mg TF
Acute-716 mg TF
Chronic-v1
Chronic-v2
t[2]
t[1]
R2X[1] = 0.828; R2X[2] = 0.106; Ellipse: Hotelling's T2 (95%)
SUM3_OH_PVLs
SUMHPPs
SUM4_OH_PVLs
SUM34_diOH_PVLs
p[2]
p[1]
R2X[1] = 0.828; R2X[2] = 0.106

E

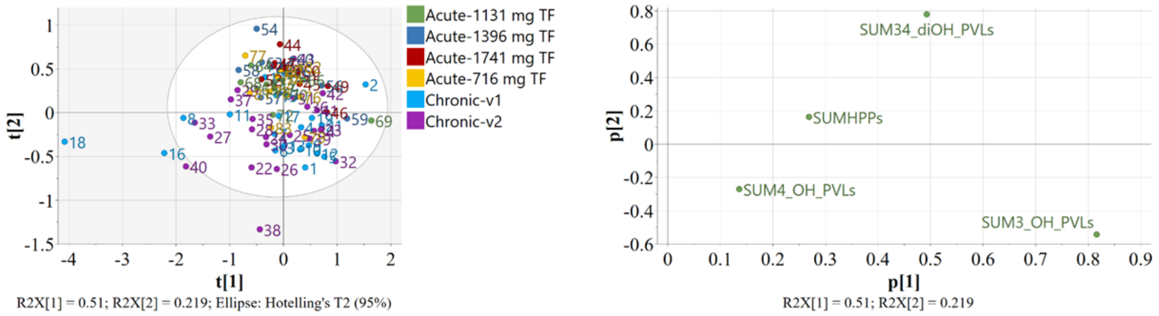
Acute-1131 mg TF
Acute-1396 mg TF
Acute-1741 mg TF
Acute-716 mg TF
Chronic-v1
Chronic-v2
t[2]
t[1]
R2X[1] = 0.51; R2X[2] = 0.219; Ellipse: Hotelling's T2 (95%)
SUM34_diOH_PVLs
SUMHPPs
SUM4_OH_PVLs
SUM3_OH_PVLs
p[2]
p[1]
R2X[1] = 0.51; R2X[2] = 0.219

F

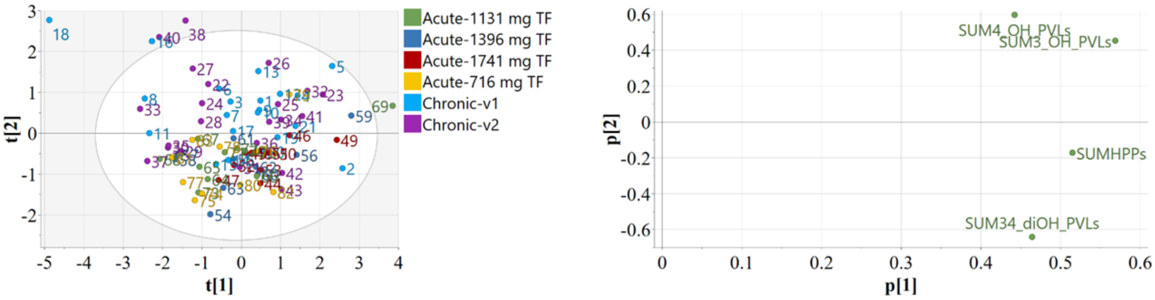

R2X[1] = 0.458; R2X[2] = 0.248; Ellipse: Hotelling's T2 (95%) R2X[1] = 0.458; R2X[2] = 0.248

G

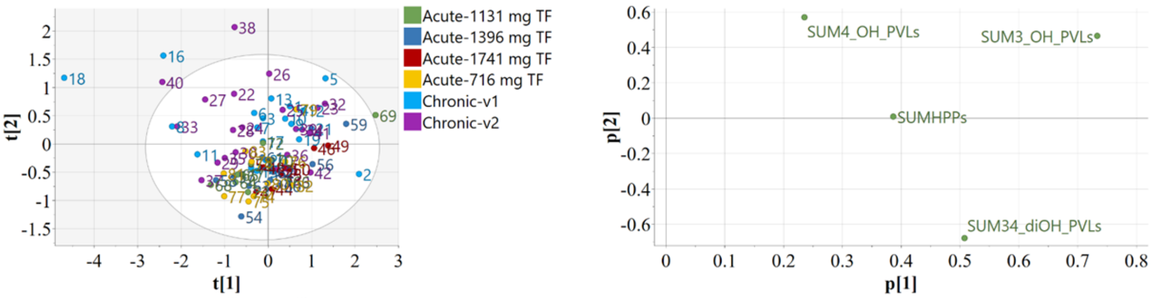

R2X[1] = 0.476; R2X[2] = 0.228; Ellipse: Hotelling's T2 (95%) R2X[1] = 0.476; R2X[2] = 0.228

H

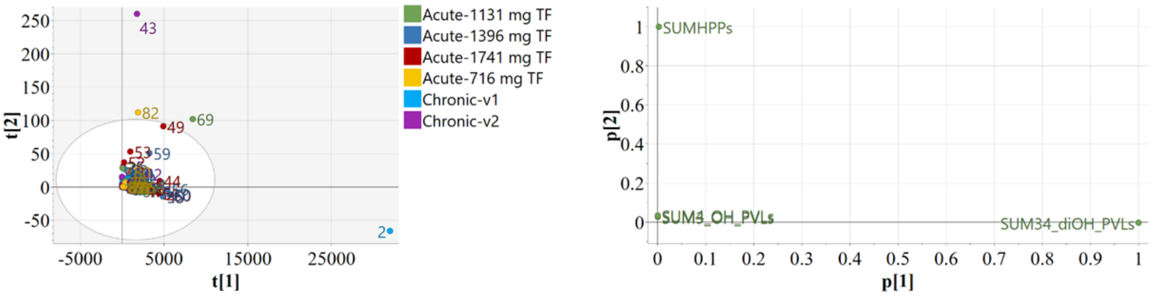

R2X[1] = 1; R2X[2] = 8.51e-05; Ellipse: Hotelling's T2 (95%) R2X[1] = 1; R2X[2] = 8.51e-05

I

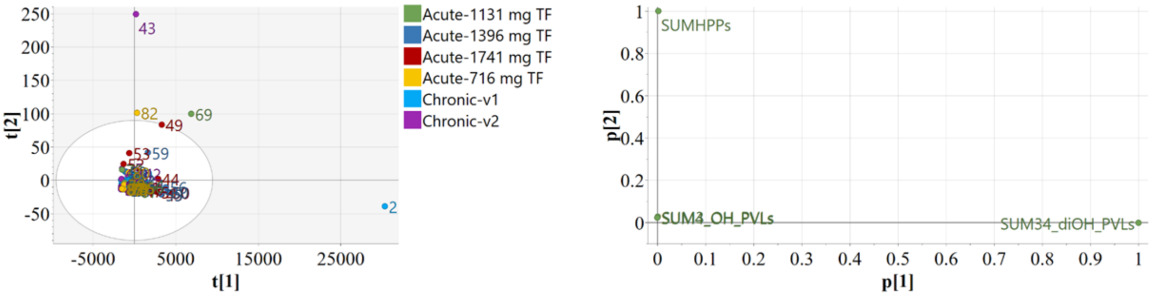

R2X[1] = 1; R2X[2] = 9.01e-05; Ellipse: Hotelling's T2 (95%) R2X[1] = 1; R2X[2] = 9.01e-05

L

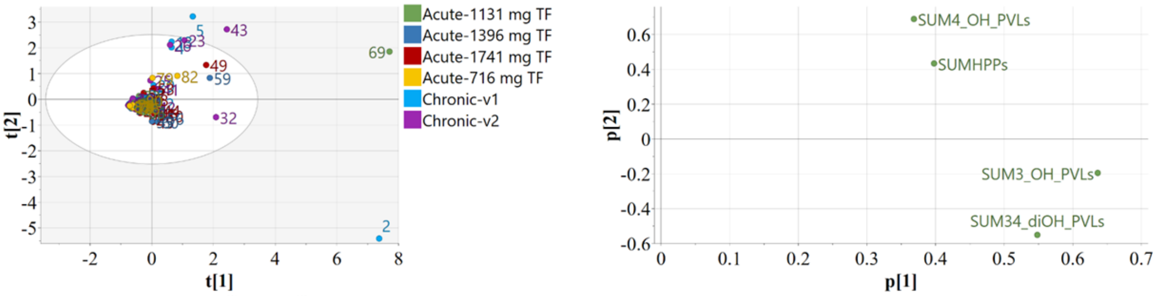

R2X[1] = 0.469; R2X[2] = 0.251; Ellipse: Hotelling's T2 (95%) R2X[1] = 0.469; R2X[2] = 0.251

M

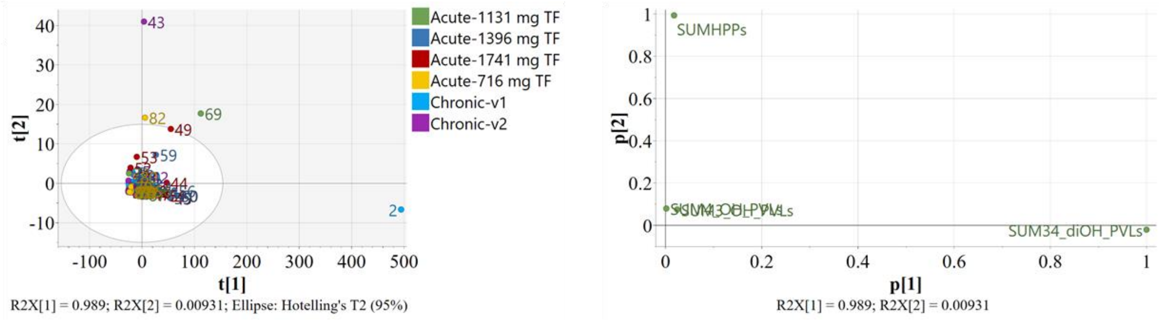

Figure S2A-M. PCA models (score and loading plots) for sums of metabolites belonging to the same aglycone compound on: (**A**) non-transformed non-scaled data, (**B**) non-transformed autoscaled data, (**C**) non-transformed centered and Pareto scaled data, (**D**) log-transformed non-scaled data, (**E**) log-transformed centered data, (**F**) log-transformed autoscaled data, (**G**) log-transformed centered and Pareto scaled data, (**H**) power-transformed non-scaled data, (**I**) power-transformed centered data, (**L**) power-transformed autoscaled data, (**M**) power-transformed centered and Pareto scaled data.

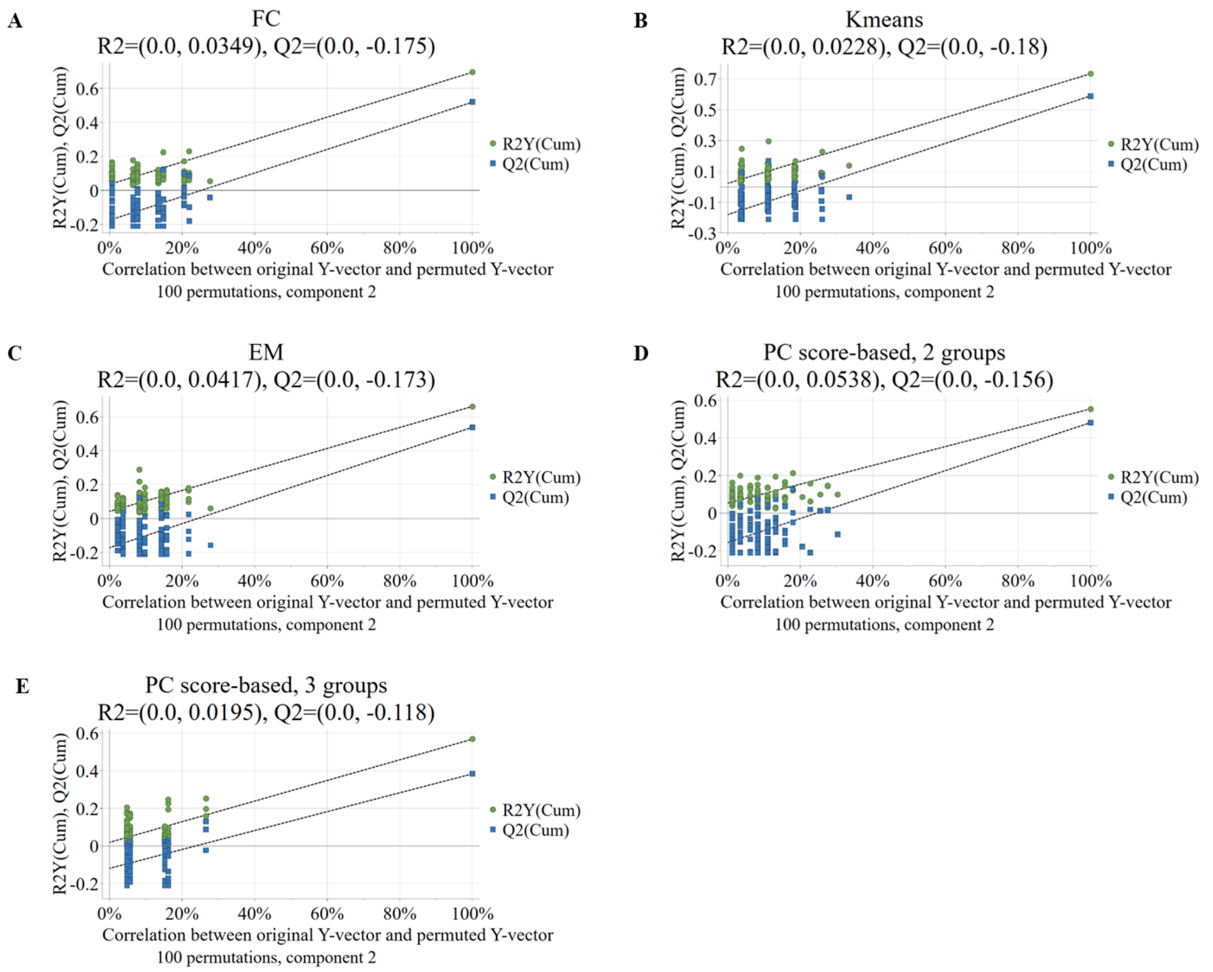

Figure S3A-E. Permutation plots of the PLS-DA models considering individual metabolites and the clusters obtained from different clustering methods: (**A**) final consensus – FC –, (**B**) k-means – Kmeans –, (**C**) expectation-maximization – EM – and PC score-based models for 2 (**D**) or 3 (**E**) groups.

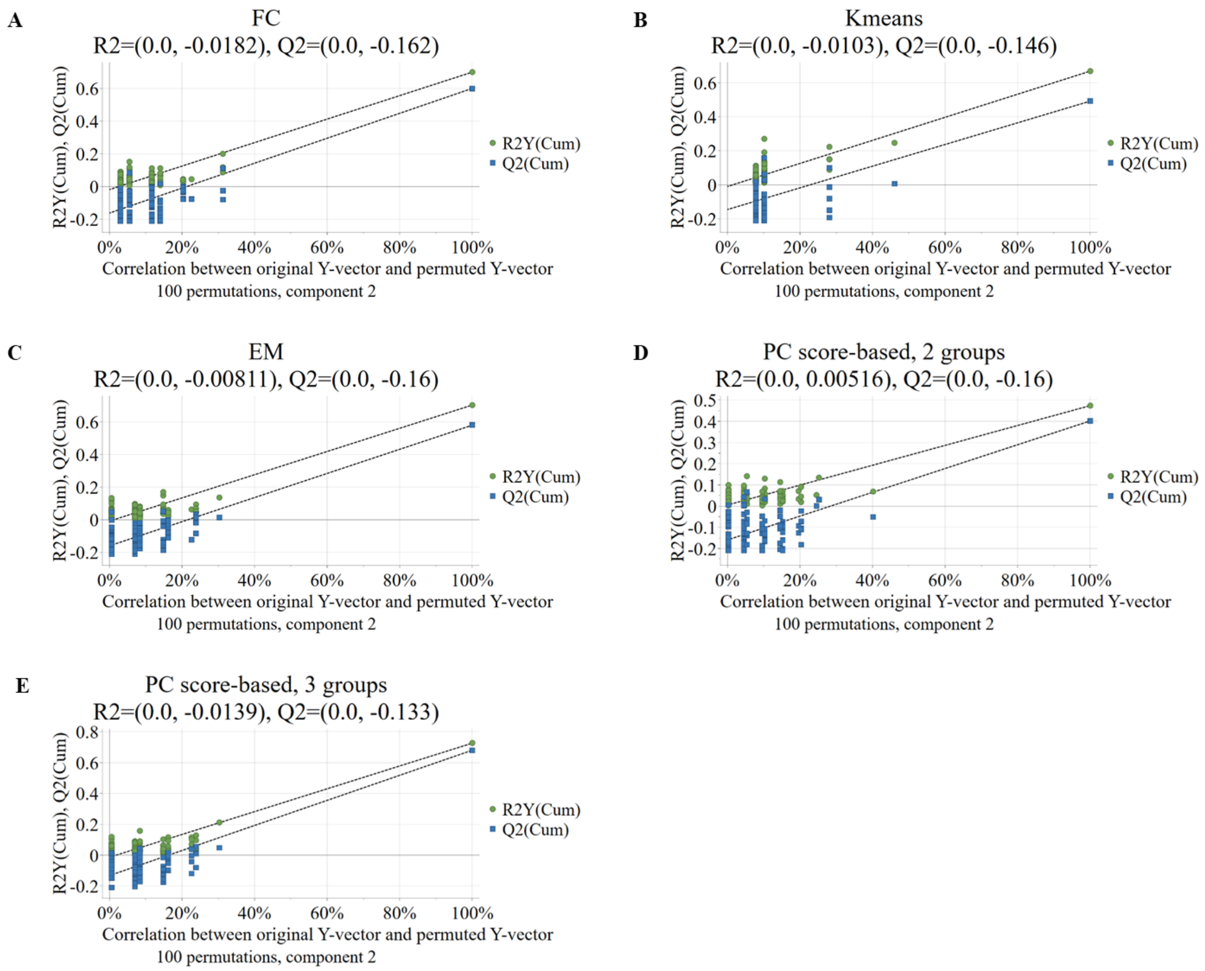

Figure S4A-E. Permutation plots of the PLS-DA models considering sums of metabolites belonging to the same aglycone family and the clusters obtained from different clustering methods: (**A**) final consensus – FC –, (**B**) k-means – Kmeans –, (**C**) expectation-maximization – EM – and PC score-based models for 2 (**D**) or 3 (**E**) groups.

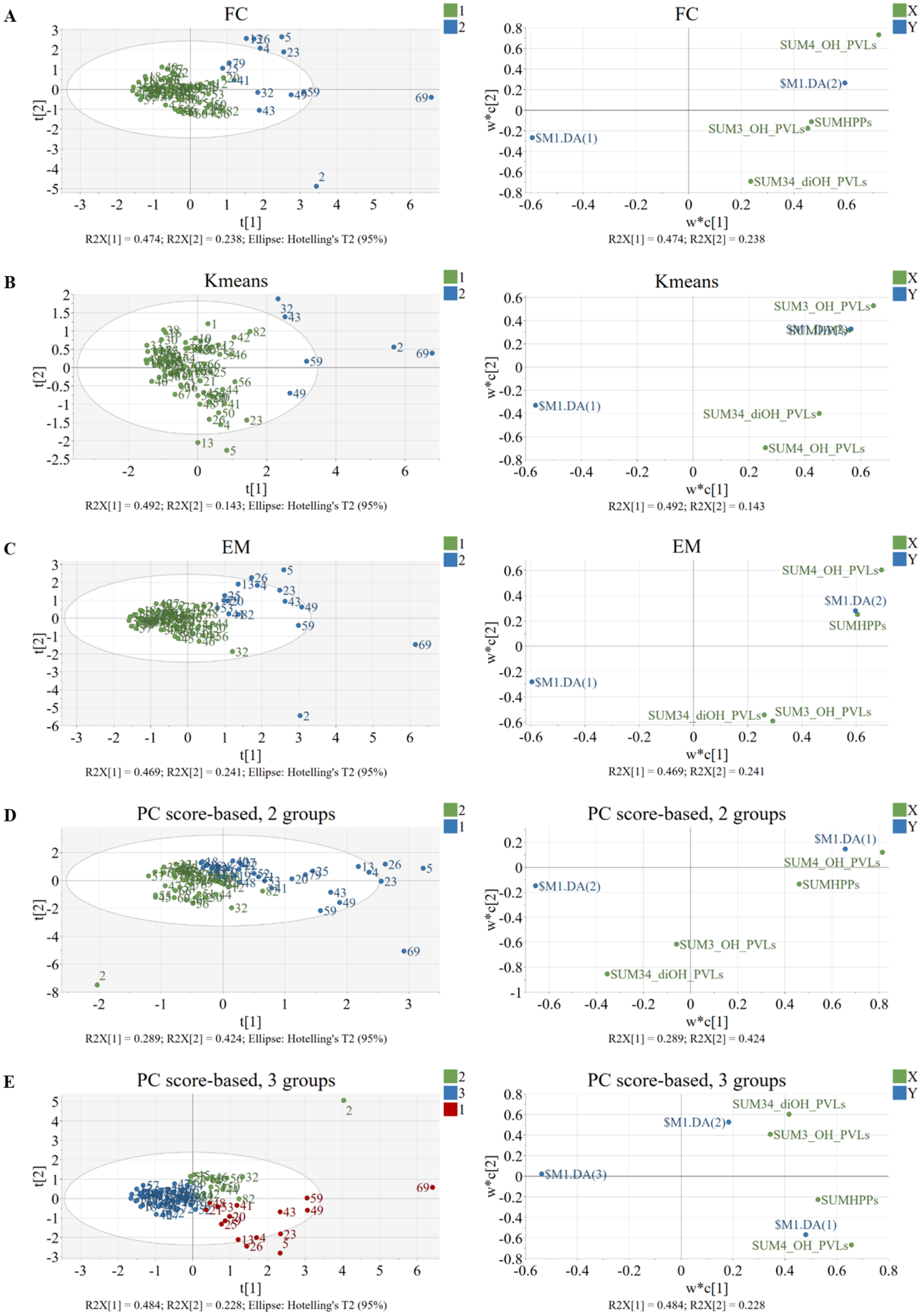

Figure S5A-E. PLS-DA models (score and loading plots) considering sums of metabolites belonging to the same aglycone family and the clusters obtained from different clustering

methods: (**A**) final consensus – FC –, (**B**) k-means – Kmeans –, (**C**) expectation-maximization – EM – and PC score-based models for 2 (**D**) or 3 (**E**) groups.

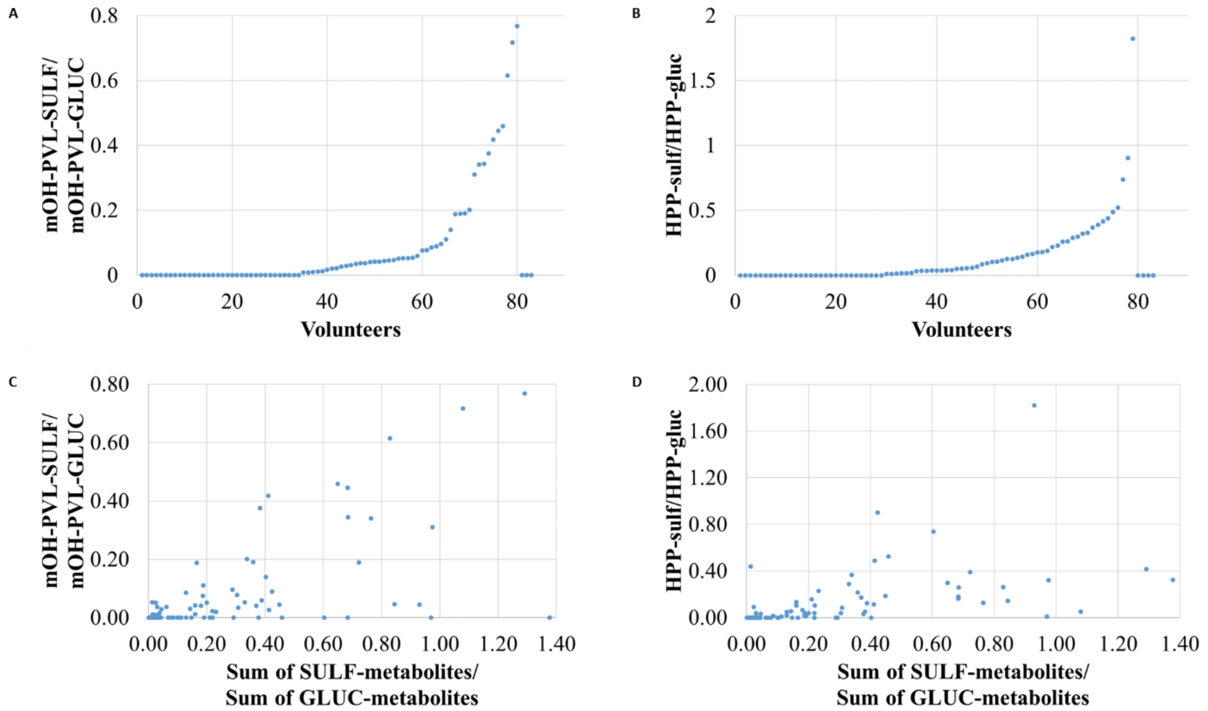

Figure S6. Inter-individual variability in phase II metabolism illustrated by the sulfate (SULF)/glucuronide (GLUC) ratio of 5-(monohydroxyphenyl)-γ-valerolactones (monoOH-PVLs) (**A**) and 3-(hydroxyphenyl)propanoic acids (HPPs) (**B**) in urine samples. Relation between the ratio of the sums of all the sulfate or glucuronide metabolites values and the sulfate/glucuronide ratio of 5-(monohydroxyphenyl)-γ-valerolactones (**C**) or 3-(hydroxyphenyl)propanoic acids (**D**).

Table S1. Classification in groups according to every clustering method (Excel file)

Table S2. VIP values for individual metabolites and aglycone compounds of each PLS-DA model considering classes defined by each clustering method selected (final consensus – FC – , k-means – Kmeans –, expectation-maximization – EM – and PC score-based models for 2 or 3 groups).

**Metabolites**	**VIP value**				
	**FC**	**Kmeans**	**EM**	**PC score-based, 2groups**	**PC score-based, 3groups**
**5-Phenyl-γ-valerolactone-3′-glucuronide**	0.80	0.83	0.66	0.82	0.71
**5-Phenyl-γ-valerolactone-3′-sulfate**	0.78	0.78	0.63	0.79	0.96
**5-Phenyl-γ-valerolactone-4′-glucuronide**	0.31	0.31	**1.36**	**1.52**	0.69
**5-(3′,4′-Dihydroxyphenyl)-γ-valerolactone**	**1.01**	**1.05**	0.59	**1.36**	**1.22**
**5-(4′-Hydroxyphenyl)-γ-valerolactone-3′-glucuronide**	**1.03**	**1.00**	0.62	**1.02**	**1.03**
**5-(3′-Hydroxyphenyl)-γ-valerolactone-4′-glucuronide**	**1.04**	0.94	0.68	0.94	**1.11**
**5-(Hydroxyphenyl)-γ-valerolactone-sulfate (3′,4′ isomers)**	**1.34**	**1.37**	0.85	0.74	**1.15**
**5-Phenyl-γ-valerolactone-sulfate-glucuronide isomer (3′,4′)**	**1.11**	**1.09**	0.70	0.29	0.96
**5-Phenyl-γ-valerolactone-methoxy-sulfate isomer (3′,4′)**	**1.08**	**1.09**	0.83	0.22	0.91
**3-Phenylpropanoic acid sulfate**	**1.32**	**1.33**	0.92	0.50	**1.09**
**3-Phenylpropanoic acid glucuronide**	0.76	0.77	**2.09**	**1.63**	**1.03**
**3′OH-PVLs**	0.88	**1.26**	0.70	0.35	0.7
**4′OH-PVLs**	**1.42**	0.65	**1.34**	**1.56**	**1.25**
**3′,4′diOH-PVLs**	0.64	0.91	0.63	0.79	**1.04**
**HPPs**	0.90	**1.08**	**1.14**	0.90	0.87

3′OH-PVLs, sum of conjugates from the aglycone 5-(3′-hydroxyphenyl)-γ-valerolactone; 4′OH-PVLs, 5-(4′-hydroxyphenyl)-γ-valerolactone; 3′,4′diOH-PVLs, 5-(3′,4′-dihydroxyphenyl)-γ-valerolactone; HPPs, 3-(hydroxyphenyl)propanoic acid.
